# Glutathione as a taste modulator: molecular mechanisms of interaction with umami and sweet taste receptors

**DOI:** 10.1016/j.fochms.2025.100319

**Published:** 2025-10-26

**Authors:** Clémence Cornut, Adeline Karolkowski, Maxence Lalis, Antoine Thomas, Rudy Menin, Jérémie Topin, Loïc Briand, Christine Belloir

**Affiliations:** a Université Bourgogne Europe, Institut Agro, CNRS, INRAE, UMR CSGA, 21000 Dijon, France; b Biospringer By Lesaffre, F-94700 Maisons-Alfort, France; c Institut de Chimie de Nice, Université Côte d'Azur, UMR 7272 CNRS, 06108 Nice, France

**Keywords:** Kokumi, G protein-coupled receptor, Ribonucleotide, TAS1R1/TAS1R3, Taste receptor, L-glutamic acid (PubChem CID: 33032), Monosodium glutamate (PubChem CID: 23672308), Inosine 5′-monophosphate disodium salt hydrate (PubChem CID: 135565601), Guanosine 5′-monophosphate disodium salt (PubChem CID: 135414246), Reduced L-glutathione (PubChem CID: 124886), Oxidized glutathione (PubChem CID: 65359), γ-glutamylcysteine (PubChem CID: 123938), S807 (N-(heptan-4-yl)benzo[d][1,3]dioxole-5-carboxamide) (PubChem CID: 22831877), Sucralose (PubChem CID: 71485)

## Abstract

Reduced L-glutathione (GSH) is a kokumi active tripeptide that enhances umami, salty, and sweet taste perceptions, probably via the calcium-sensing receptor (CaSR). In this study, we report that GSH is a partial agonist of the umami taste receptor (hTAS1R1/rTAS1R3). Using cellular assays, we revealed synergistic effects of GSH with L-glutamic acid (L-Glu) but not with 5′-ribonucleotides. Combining molecular modeling and mutagenesis studies, we mapped the GSH binding site located between the two lobes of the Venus Flytrap domain (VFT) of hTAS1R1. Interestingly, GSH is a weak agonist of the sweet taste receptor (hTAS1R2/hTAS1R3) and synergizes with sucralose via the rTAS1R3 subunit. Using the chimeric TAS1R3 receptor and site-directed mutagenesis, we showed that GSH binds to TAS1R3-VFT. This research provides increased understanding of the molecular interactions between GSH and TAS1Rs and suggests that the kokumi activity of GSH is more complex than affecting CaSR alone.

## Highlights

• Reduced L-glutathione (GSH) is a partial agonist of hTAS1R1/rTAS1R3.
• GSH synergizes with L-Glu but not with 5′-ribonucleotides.
• GSH binds to hTAS1R1-VFT at a binding site adjacent to that of L-Glu.
• GSH is a weak agonist of hTAS1R2/hTAS1R3 and hTAS1R2/rTAS1R3.
• GSH synergizes with sucralose in affecting of rTAS1R3-VFT.

## Graphical abstract

**Figure:**
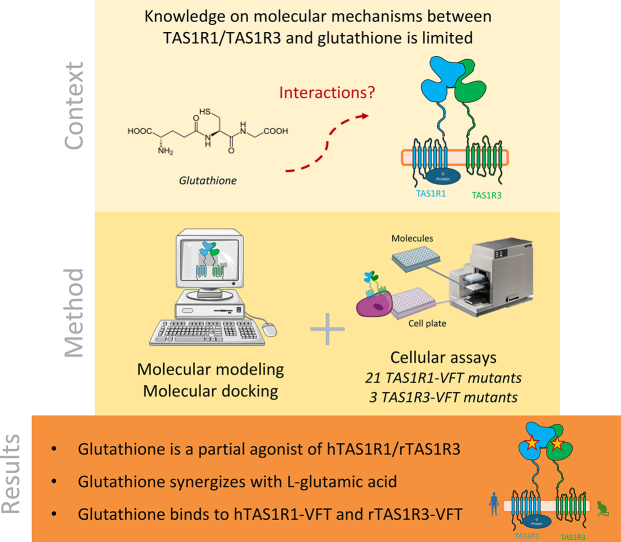

## 1 Introduction

Reduced L-glutathione (GSH, γ-L-glutamyl-L-cysteinyl-glycine) is a γ-glutamyl tripeptide that is abundantly present in many food products, including fruits, vegetables, raw meat, dairy products, cereals, and yeast extracts (Schroeder et al., 1939; Ueda et al., 1997; Wierzbicka et al., 1989). GSH and γ-L-glutamyl peptides are typical kokumi molecules, which are usually tasteless (instead providing continuity, mouthfulness, and thickness) but enhance the perception of salty, sweet, and umami tastes (Ohsu et al., 2010; Ueda et al., 1997). The homodimeric calcium-sensing receptor (CaSR) is a candidate taste receptor for kokumi in humans and animals (including mice and cats) (Amino et al., 2016; Goralski & Ram, 2022; Kitajima et al., 2023; Maruyama et al., 2012; Ohsu et al., 2010; Wang et al., 2006; Yamaguchi et al., 2025; Yamamoto et al., 2020) and is expressed in taste papillae (Maruyama et al., 2012). GSH has been shown to be a positive allosteric modulator (PAM) of CaSR with in vitro activation, which is consistent with the kokumi taste intensities evaluated by human sensory analysis (Li et al., 2022; Ohsu et al., 2010).

Umami and sweet tastes are detected by type 1 receptors (TAS1Rs), which are class C G-protein coupled receptors (GPCRs). The heterodimeric complex TAS1R1/TAS1R3 mediates the detection of umami compounds, whereas the TAS1R2 and TAS1R3 subunits assemble to form the sweet taste receptor (Belloir et al., 2017; Chandrashekar et al., 2006; Li et al., 2002; Nelson et al., 2002). The class C GPCR family includes metabotropic glutamate receptors (mGluRs), γ-aminobutyric acid B-type receptors (GABA_B_Rs), CaSR, and several orphan receptors (Brauner-Osborne et al., 2007; Chavez-Abiega et al., 2020; Ohsu et al., 2010; Pin et al., 2003). TAS1Rs share the architecture of class C GPCRs and possess a large extracellular domain composed of a Venus flytrap domain (VFT) and a cysteine-rich domain (CRD) (except in GABA_B_R subunits, which do not have a CRD) linked to the transmembrane domain (TMD) (Chun et al., 2012). Structural studies conducted via X-ray crystallography and cryo-electron microscopy (cryo-EM) have demonstrated that the VFT consists of two lobes separated by a large cleft, forming the orthosteric binding site for ligands (Acher et al., 2022; Du et al., 2021; Geng et al., 2016; Koehl et al., 2019; Krishna Kumar et al., 2024; Kunishima et al., 2000; Lebon, 2023; Lin et al., 2021; Muto et al., 2007; Nasrallah et al., 2021; Seven et al., 2021). The recent determination of the cryo-EM structure of human TAS1R2/TAS1R3 (hTAS1R2/hTAS1R3) provided a major breakthrough (Juen et al., 2025; Shi et al., 2025; Wang et al., 2025). The structures of TAS1R2/TAS1R3, solved in the apo state and bound to two artificial sweeteners, sucralose and aspartame, have provided insights into its mechanism of activation. The TAS1R2/TAS1R3 receptor was revealed to have an asymmetric heterodimeric organization, with the TAS1R2 subunit functioning as the exclusive ligand-binding site for sucralose and aspartame. The binding of agonist sweeteners to VFT results in a moderate conformational change in TAS1R2-VFT, which triggers a conformational cascade involving a VFT shift and CRD bending. Interactions with VFT have been shown to induce a major conformational change in the intracellular region of TAS1R2-TMD, which triggers signaling cascades (Juen et al., 2025; Shi et al., 2025; Wang et al., 2025). In contrast, TAS1R3-VFT has been shown to adopt a more open conformation with no additional ligand density identified. This observation contrasts with the results of biochemical studies showing the ability of TAS1R3-VFT to interact with several sweet and umami compounds (Belloir et al., 2025; Laffitte et al., 2022; Maîtrepierre et al., 2012; Nie et al., 2005).

Potentiation by two 5′-ribonucleotides, inosine 5′-monophosphate (IMP) and guanosine 5′-monophosphate (GMP), is a hallmark of umami taste (Kuninaka, 1960, Kuninaka, 1964; Yamaguchi, 1991). The monosodium glutamate (MSG) sensory recognition threshold decreases in the presence of 0.05 mM IMP from 7.66 to 0.20 mM (Shigemura et al., 2009). Functional expression assays have shown that hTAS1R1/hTAS1R3 responds to various umami compounds, including L-amino acids (mainly L-glutamic acid (L-Glu) and, to a lesser extent, L-aspartic acid (L-Asp)), small peptides and their derivatives, and high-potency natural and synthetic compounds, with dose-responses consistent with their umami potencies (Li et al., 2002; Nelson et al., 2002; Servant & Frerot, 2022; Toda et al., 2013; Zhang et al., 2008; Zhao et al., 2003). The response of TAS1R1/TAS1R3 to L-Glu is enhanced by 5′-ribonucleotides (Li et al., 2002; Servant & Frerot, 2022; Shigemura et al., 2009). The site-directed mutagenesis and chimeric mouse-human receptors, combined with molecular modeling and cell-based assays demonstrated that the VFT of TAS1R1 contains the orthosteric binding site of the umami receptor (Li et al., 2002; Toda et al., 2013; Xu et al., 2004; Zhang et al., 2008). Additional binding sites have been identified in synergy or inhibition. For example, TAS1R1-TMD interacts with methional and S807 (N-(heptan-4-yl)benzo[d][1,3]dioxole-5-carboxamide) (Toda et al., 2013; Zhang et al., 2008), whereas cyclamate and clofibrate act as an enhancer and as an inhibitor of TAS1R1/TAS1R3, respectively (Kochem & Breslin, 2017; Zhang et al., 2008). The molecular mechanism of synergy with ribonucleotides involves a cooperative model in which L-Glu binds to the hinge region of TAS1R1-VFT, inducing VFT closure, whereas IMP binds to an adjacent site, stabilizing the closed conformation of TAS1R1-VFT (Zhang et al., 2008).

While tasteless, GSH has also been reported to moderately activate the umami TAS1R1/TAS1R3 receptor with an EC_50_ value of 11.3 mM, which is consistent with its weak umami taste intensity, as evaluated by sensory tests (Broadhead et al., 2011; Servant & Frerot, 2022). A concentration of 3.26 mM GSH elicits an umami perception equivalent to 0.05 % MSG (2.96 mM) (Servant & Frerot, 2022).

Here we report the identification of the ligand-binding sites for GSH in the umami hTAS1R1/rTAS1R3 taste receptor. Combining molecular modeling, chimeric receptor, mutagenesis studies, and in vitro cell-based assays, we showed that GSH binds to hTAS1R1-VFT at a binding site adjacent to those of L-Glu and IMP. Interestingly, we also demonstrated that GSH interacts with rTAS1R3-VFT.

## 2 Materials and methods

### 2.1 Chemical compounds

L-Glu (≥ 98 %), L-cysteine (L-Cys, ≥98 %), glycine (Gly, ≥98 %), GSH (≥98 %), oxidized L-glutathione (GSSG, 98 %), L-glutamate monosodium salt (MSG, ≥98 %), inosine 5′-monophosphate disodium salt hydrate (IMP, ≥99 %), guanosine 5′-monophosphate disodium salt hydrate (GMP, ≥99 %), S807 (95 %), and sucralose (99.6 %) were purchased from Sigma-Aldrich (Merck Group, Saint-Quentin-Fallavier, France). γ-L-Glutamyl-L-cysteine (Glu-Cys, 95.2 %) was purchased from MedChemExpress (Monmouth Junction, NJ, USA).

### 2.2 Molecular modeling and molecular docking

The predicted TAS1R1 structures were generated via AlphaFold2 (Jumper et al., 2021) using mGluR1 (PDB ID: 1EWK) as a template. Multiple sequence alignments (MSAs) were created via jackHHMER on uniref90 and via Mgnify and Hhblits on BFD and Uniclust30. The hTAS1R2/hTAS1R3 structure was used as a template. Five models were produced and subjected to relaxation and energy minimization steps. Using the pLDDT metric and the RMSD of the backbone of the cryo-EM structure of TAS1R2/TAS1R3 (6.033 Å), the best of these five models was selected for further investigation.

The model was aligned with the experimental structure of hTAS1R2/hTAS1R3 (PDB ID: 9nor) to locate the binding pocket, which was identified on the basis of the position of L-Glu. Pocket identification and description were performed via MDPocket detection software (https://github.com/Discngine/fpocket) (Schmidtke et al., 2011).

Molecular docking was performed using AutoDock Vina (v1.2.5) (https://github.com/ccsb-scripps/AutoDock-Vina) (Eberhardt et al., 2021). Proteins and ligands were prepared via the MGLTools Python script package. Docking grids were designed to contain all the residues identified by MDPocket binding pocket detection software. Exhaustiveness was set to 50, and the number of random seeds was set to 123. All the other parameters were kept at their default values. To confirm the reliability of the docking protocol, a re-docking experiment was performed on the same mGluR1 structure (PDB ID: 1EWK) with L-Glu, which successfully reproduced the experimental cryo-EM binding pose with an RMSD of 1.292 Å **(Fig. S1**).

The same procedure was used to dock GSH inside hTAS1R3-VFT.

### 2.3 Constructs for chimeric Gα15i2 and TAS1R receptors

Chimeric Gα proteins, such as Gα16Gust44, Gα16Gi3 or Gα15i2, are widely used in the functional expression of TAS1R receptors (Backes et al., 2015; Suess et al., 2015; Toda et al., 2021, Toda et al., 2013, Toda et al., 2018; Zhang et al., 2008). Chimeric Gα15i2 ((Toda et al., 2013), **Supplementary data**) was generated by replacing the last five residues of the C-terminal tail of rat Gα15i2 (EINLL) (UniProt number G3V6N8; https://www.uniprot.org/uniprot/) with the counterpart sequence from human GNAI2 (DCGLF) (UniProt number P04899-1) and cloning into the pcDNA3.1/Hygro vector (Invitrogen, Thermo Fisher Scientific, Illkirch, France) between the *Hind*III and *Not*I restriction sites, resulting in the plasmid pcDNA3.1-Gα15i2. Chimeric Gα16Gust44 construct have already been reported (Raliou et al., 2011). Briefly, Gα16Gust44 was generated by replacing the C-terminal of Gα16 with the C-terminal 44 residues of gustducin. As previously described, hTAS1R1 was amplified from human fungiform papillae cDNA and cloned and inserted into pcDNA3 (Invitrogen), generating pcDNA3-TAS1R1-WT (wild-type) (Raliou et al., 2011). Whole-plasmid sequencing (Genewiz, Azenta Life Sciences, Leipzig, Germany) confirmed the sequence corresponding to variant 2 (NM_138697.4; UniProtKB number Q7RTX1–1). hTAS1R1 was co-expressed with rat TAS1R3 (rTAS1R3) (UniProtKB number Q923K1) instead of hTAS1R3. The cDNA coding region of rTAS1R3 was synthesized with optimized-codon usage for human expression (Genewiz) and was cloned and inserted into the mammalian pcDNA4/myc-HisA (Invitrogen) expression vector between the *Eco*RI and *Not*I restriction sites, resulting in the plasmid pcDNA4-rTAS1R3. Single-point mutants of the TAS1R1 and TAS1R3 subunits were constructed on the basis of pcDNA3-hTAS1R1 and pcDNA4-rTAS1R3 via site-directed mutagenesis (Genewiz). To identify the binding site of GSH in TAS1R3, we constructed human/rat chimeras swapping the extracellular domain (including CRD and VFT) and TMD of the two species. The sequence integrity of all the plasmid constructs was verified via automated DNA sequencing (Genewiz). In addition, eight TAS1R1-VFT point mutations were chosen as they had previously been involved in L-Glu and IMP binding: H71A, S172A, D192A, Y220A, R277A, E301A, S306A, and H308A (Toda et al., 2013; Zhang et al., 2008). Then, thirteen mutants were synthesized based on GSH molecular docking resulting in mutation: H71Y, H71S, H71L, D147A, R151A, A170W, D192F, D192E, L223A Q278A, Q278K, Q278Y and A302W.

### 2.4 Taste receptor functional assays

HEK293T/17 cells (ATCC CRL-11268) were seeded into clear bottom black 96-well plates coated with poly-d-lysine at a density of 32500 cells/well in 100 μL of DMEM containing 4.5 g/L glucose and supplemented with penicillin/streptomycin, 2 mM GlutaMAX and 10 % dialyzed fetal bovine serum. The plates were incubated for 24 h at 37 °C and 7.3 % CO_2_ in a humidified atmosphere. After incubation, using Fugene® 4 K (0.46 μL/well, Promega, Illkirch, France), the cells were transiently transfected with pcDNA3-hTAS1R1-WT (60 ng/well) or TAS1R1 mutants, pcDNA4-rTAS1R3 (60 ng/well), pcDNA3-Gα15i2 (10 ng/well), and pGP-CMV-GCaMP6s (Addgene #40753; 10 ng/well) as a genetically encoded calcium indicator. Twenty-four hours after transfection, the HEK293T/17 cells were rinsed with 100 μL of C1 buffer (130 mM NaCl, 5 mM KCl, 10 mM HEPES, 2 mM CaCl_2_, and 5 mM sodium pyruvate, pH 7.4). For each molecule tested, a semi-logarithmic concentration range was created consisting of seven increasing concentrations and a negative control (zero dose) made of buffer C1. Binary mixtures with different molecules were also made to study potential synergy. In this case, the concentration range was tested in C1 buffer containing the other molecules to be tested. The concentration ranges varied depending on the molecule, its solubility, and its effectiveness in activating the receptor. All the solutions were made on the day of the test to prevent any oxidation of the compounds. If needed, the pH of each solution was adjusted by to pH 7.4 by adding NaOH. After 20 s of basal fluorescence calcium measurement, the cells were stimulated via automatic injection of a range of compounds in eight wells, and the signal emission was recorded for an additional 70 s, via a FlexStation 3® fluorometric microplate reader (Molecular Devices, San Jose, CA, USA). As a control, molecules were also tested on mock-transfected cells with the empty vector. The emitted fluorescence was measured at a wavelength of 510 nm after excitation at 488 nm. Each measurement was performed in duplicate on the same plate, and the experiments were carried out at least four times. Kinetic data were acquired via SoftMax Pro 5.4.6 software (Molecular Devices). For each well, the decrease in the raw fluorescence as the relative fluorescence changed was calculated as ΔF/F0. Replicates were averaged for the wells subjected to the same stimuli, the background signal was subtracted, and the fluorescence changes in mock-transfected cells were subtracted. The half-maximal effective concentration (EC_50_) and maximal signal amplitude (max ∆F/F0) were calculated via non-linear regression of the sigmoidal function four-parameter logistic equation [f(x) = min + (max − min)/(1 + (x/EC_50_)^nH^)] via SigmaPlot 15.0 software (Grafiti LLC, Palo Alto, CA, USA). Statistical data analysis, notably one-way analysis of variance (ANOVA) with Dunnett's test, was performed via XLSTAT 2023 (Lumivero, Denver, CO, USA). For a clearer understanding of the results, the amplitude values in certain dose-response curves were normalized to max ∆F/F0 = 1, to eliminate the differences in amplitude.

## 3 Results and discussion

### 3.1 GSH is a partial agonist of the umami taste receptor

We first investigated whether GSH activated the human umami taste receptor. To increase umami receptor activity to measurable levels, we co-expressed hTAS1R1 with rTAS1R3 instead of hTAS1R3 (Festring & Hofmann, 2011; Suess et al., 2014; Zhang et al., 2008). Given that umami compounds, including amino acids and nucleotide derivatives, are known to interact with the TAS1R1 subunit of the umami heterodimeric receptor (Toda et al., 2013; Zhang et al., 2008), the hTAS1R1/rTAS1R3 receptor assay is likely to resemble the detection of umami compounds in human taste receptor cells. To this end, hTAS1R1/rTAS1R3 and the chimeric G protein Gα15i2 were transiently expressed in HEK293T cells. Cellular responses were assessed via calcium imaging using a fluorescent protein calcium biosensor (GCaMP6s) and compared with those of mock-transfected cells.

**Fig. 1:**
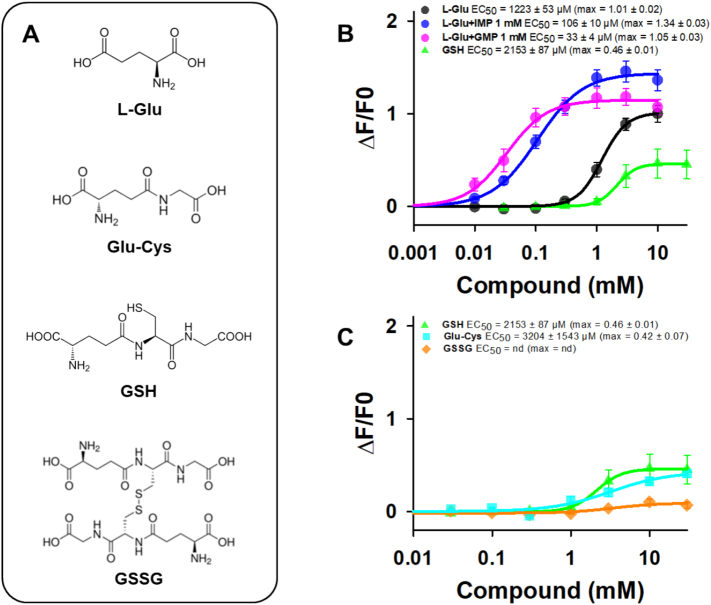
Chemical structures of L-Glu, Glu-Cys, GSH, and GSSG (A), and dose-response curves of hTAS1R1/rTAS1R3 stimulated with L-Glu, L-Glu + 1 mM IMP, L-Glu + 1 mM GMP, GSH (B) and GSH, Glu-Cys, and GSSG (C). The data are presented as the mean ± sem of 8 wells from 4 independent experiments. L-Glu: L-glutamic acid; Glu-Cys: γ-L-glutamyl-L-cysteine; GSH: reduced L-glutathione; GSSG: oxidized L-glutathione; IMP: inosine 5′-monophosphate; GMP: guanosine 5′-monophosphate; nd: not determined.

We found that L-Glu and GSH elicited dose-dependent hTAS1R1/rTAS1R3 activation, leading to EC_50_ values of 1223 ± 53 μM and 2153 ± 87 μM (Fig. 1), respectively, in agreement with previously reported data (Li et al., 2002; Servant & Frerot, 2022; Zhang et al., 2008). Interestingly, we observed that GSH activated hTAS1R1/rTAS1R3 with less efficacy, leading to a maximal GSH response (max ∆F/F0 = 0.46 ± 0.01) that was 2.2-fold lower than that elicited by L-Glu (max ∆F/F0 = 1.01 ± 0.02) (Fig. 1B). We then tested whether the reduced dipeptide Glu-Cys, the precursor of GSH, activated the hTAS1R1/rTAS1R3 receptor. We found that the application of Glu-Cys produced weak agonist activity, with an EC_50_ value of 3204 ± 1543 μM and a max ∆F/F0 of 0.42 ± 0.07 (Fig. 1C). As a control, the disulfide dimer GSSG did not activate hTAS1R1/rTAS1R3.  As previously reported (Li et al., 2002; Zhang et al., 2008), we confirmed the potentiation of the hTAS1R1/rTAS1R3 response to L-Glu by the 5′-ribonucleotides IMP and GMP (Fig. 1B), whereas these 5′-ribonucleotides alone did not activate hTAS1R1/rTAS1R3 **(Fig. S2**). Finally, we tested S807, an umami compound shown to interact with hTAS1R1-TMD (Zhang et al., 2008). We observed that the application of S807 produced strong agonist activity, leading to EC_50_ and max ∆F/F0 values of 1.02 ± 0.08 μM and 1.87 ± 0.03, respectively (**Fig. S2**). Taken together, our results demonstrated that GSH activated hTAS1R1/rTAS1R3 with low efficacy by acting as a partial agonist, which is consistent with its weak umami taste, as previously described by sensory analysis (Servant & Frerot, 2022).

### 3.2 Interactions between GSH and TAS1R3

#### 3.2.1 GSH activates the sweet taste receptor and interacts with TAS1R3

**Fig. 2:**
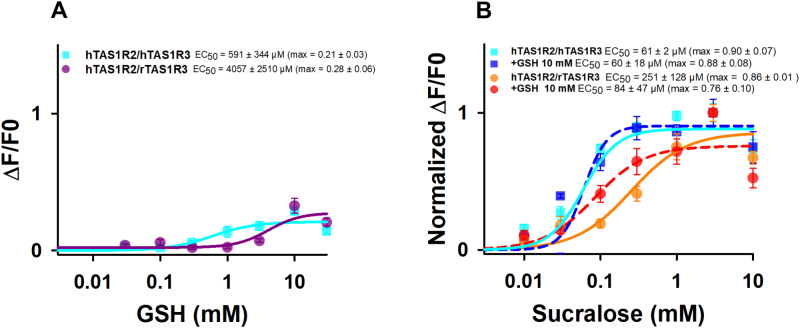
Dose-response curves of hTAS1R2/hTAS1R3 (blue curve) and hTAS1R2/rTAS1R3 (purple curve) stimulated with GSH (A), and of hTAS1R2/hTAS1R3 (blue curves) and hTAS1R2/rTAS1R3 (orange and red curves) stimulated with sucralose in the absence (full curves) or presence of 10 mM GSH (dotted curves) (B). The data are presented as the mean ± sem of 8 wells from 4 independent experiments. GSH: reduced L-glutathione. (For interpretation of the references to colour in this figure legend, the reader is referred to the web version of this article.)

As a control, we examined the calcium response of cells expressing the sweet taste receptor hTAS1R2/rTAS1R3 after stimulation with GSH and sucralose (Fig. 2A and B). Surprisingly, we observed that GSH was a weaker agonist of hTAS1R2/rTAS1R3 than of hTAS1R2/hTAS1R3, leading to EC_50_ values of 4057 ± 2510 and 591 ± 344 μM, respectively. As expected, hTAS1R2/rTAS1R3 exhibited a higher EC_50_ value (251 ± 128 μM) than hTAS1R2/hTAS1R3 for sucralose (61 ± 2 μM) revealing differences between species. Given that TAS1R2-VFT contains the major binding site for sweet compounds (Juen et al., 2025; Shi et al., 2025; Wang et al., 2025), we hypothesized that the TAS1R3 subunit is responsible for sweet-taste receptor activation by GSH. We therefore tested whether GSH could enhance the activity of hTAS1R2/rTAS1R3 and hTAS1R2/hTAS1R3 stimulated with sucralose. As shown in Fig. 2B, the presence of 10 mM GSH significantly increased the hTAS1R2/rTAS1R3 response to sucralose, resulting in a 3-fold decrease in the EC_50_ value. In contrast, the response of hTAS1R2/hTASR3 to sucralose in the presence of 10 mM GSH was unaffected. These data indicated that the rTAS1R3 subunit was the origin of the synergy between sucralose and GSH. Interestingly, kokumi compounds have been shown to increase the perception of sweetness (Ohsu et al., 2010). The synergy observed between sucralose and GSH provided further evidence supporting the usefulness of GSH to reduce sugar in foods and beverages while preserving sweetness intensity.

Finally, GSH, a kokumi molecule, was a better agonist of CaSR (EC_50_ = 0.71 μM) (Ohsu et al., 2010), compared to the EC_50_ value of 591 ± 344 μM that we measured for hTAS1R2/hTAS1R3 receptor. These data suggest that the kokumi effect was primarily mediated by CaSR activation. Nevertheless, our results demonstrated that CaSR may not be the only receptor involved in the kokumi perception.

#### 3.2.2 GSH interacts with TAS1R3-VFT

**Fig. 3:**
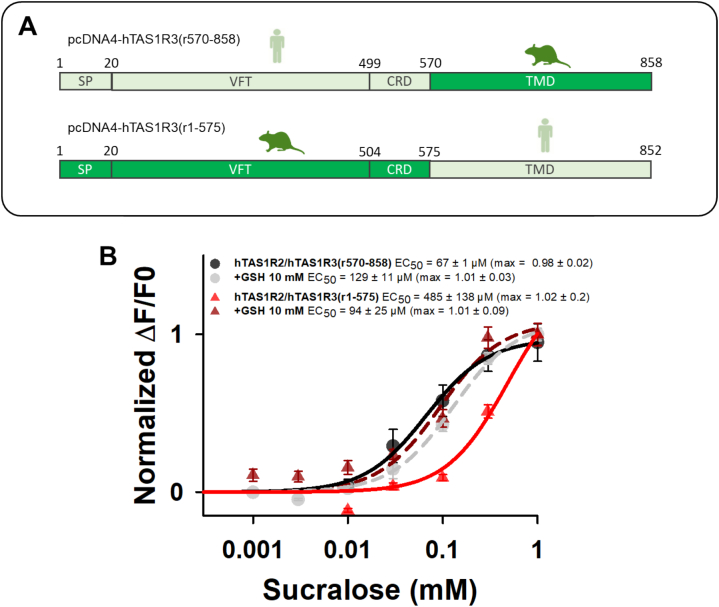
Constructs of hTAS1R2/hTAS1R3(r570–858) and hTAS1R2/hTAS1R3(r1–575) (A), and dose-response curves of hTAS1R2/hTAS1R3(r570–858) (black and gray curves) and hTAS1R2/hTAS1R3(r1–575) (red and brown curves) stimulated with sucralose in the absence (full curves) or presence of 10 mM GSH (dotted curves) (B). The data are presented as the mean ± sem of 8 wells from 4 independent experiments. SP: signal peptide; VFT: Venus flytrap domain; CRD: cysteine-rich domain; TMD: transmembrane domain; GSH: reduced L-glutathione. (For interpretation of the references to colour in this figure legend, the reader is referred to the web version of this article.)

Our data revealed that the TAS1R3 subunit was responsible for the synergy between GSH and sucralose, most likely via its VFT domain. To test this hypothesis, we generated two chimeric receptors, rTAS1R3 and hTAS1R3. hTAS1R3(r570–858) consists of the N-terminal extracellular domain of hTAS1R3 and the TMD of rTAS1R3. Conversely, hTAS1R3(r1–575) consists of the N-terminal extracellular domain of rTAS1R3 and the TMD of hTAS1R3 (Fig. 3A). By co-expressing TAS1R3 chimeras with hTAS1R2, we tested the ability of GSH to increase the activity of the chimeric receptors stimulated by sucralose. As shown in Fig. 3B, the presence of 10 mM GSH increased the response of hTAS1R2/hTAS1R3(r1–575) to sucralose, resulting in a 5.2-fold decrease in the EC_50_ value. In contrast, the response of hTAS1R2/hTAS1R3(r570–858) to sucralose in the presence of GSH was unaffected. Taken together, our results demonstrated that GSH synergized with sucralose at the level of rTAS1R3-VFT. We then examined the calcium response of cells expressing the umami receptor hTAS1R1 with chimeric TAS1R3 receptors after stimulation by L-Glu. Unfortunately, we found that hTAS1R1/hTAS1R3(r1–575) and hTAS1R1/hTAS1R3(r570–858) were not functional and did not respond to L-Glu (data not shown).

#### 3.2.3 Mapping the ligand-binding site of GSH on TAS1R3-VFT

To identify the key positions of the hTAS1R3 receptor involved in GSH recognition, we followed a mutated amino acid selection strategy based on a combination of evolutionary (conservation) and functional (activity) criteria. We first identified TAS1R1 residues known to affect agonist responses when mutated and then focused on positions that differ between hTAS1R3 and rTAS1R3. These species-specific variations could underlie the differential ligand sensitivity of hTAS1R3 and rTAS1R3. Finally, molecular docking indicated that GSH could bind to a cavity in the VFT of hTAS1R3, whose position was very close to that observed in hTAS1R1.

Following these criteria, we selected a first series of mutations around this binding pocket, namely, five residues located deep in the binding cavity that we mutated to their human counterparts (L68N, S168G, A277V, R278H, S302A) (Mut1) (**Fig. S2**). We also selected other residues surrounding the cavity that differed from species to species and were not directly involved in ligand binding but rather in dynamic phenomena resulting in signal transmission, namely, four residues located less deep in the binding cavity that we mutated to their human counterparts (Y281H, S282A, P67S, L72W) (Mut2) (**Fig. S3**). Finally, we targeted positions at the interface between the VFTs of TAS1R1 and TAS1R3. These residues, located far from the binding cavity, are unlikely to interact with GSH, therefore this mutant was selected as a negative control to probe GSH binding. A double mutant (E221Q and R173L) (Mut3) was thus created. For each group, we constructed multisite mutants (penta-, quadruple-, or double-mutants) to assess their combined effects efficiently.

**Fig. 4:**
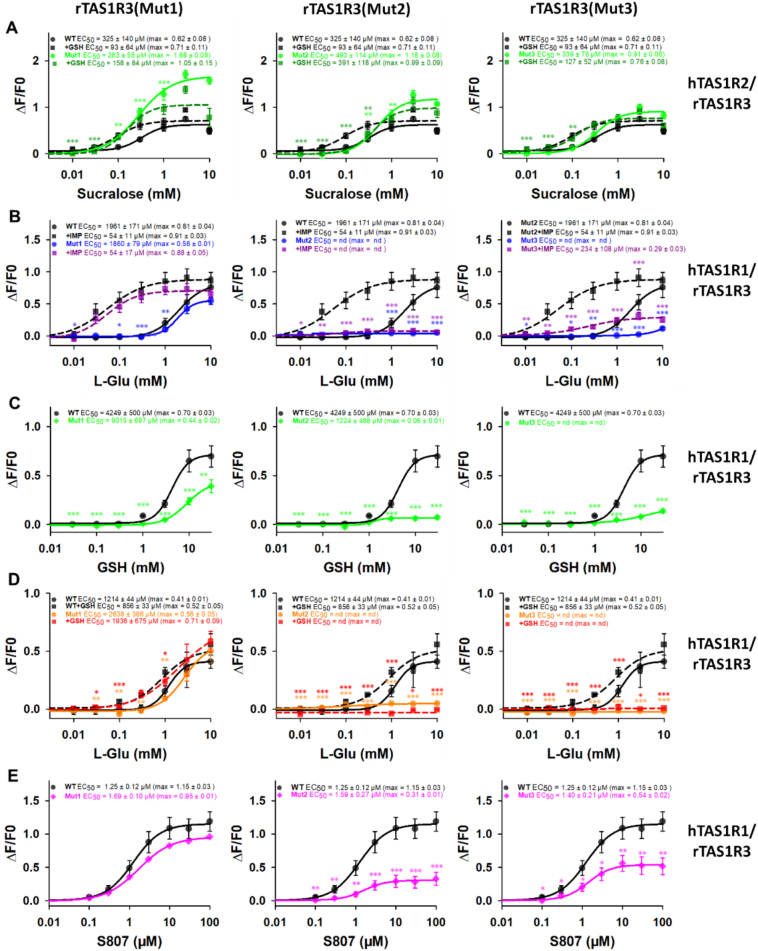
Dose-response curves of rTAS1R3(Mut1) (left), rTAS1R3(Mut2) (center), and rTAS1R3(Mut3) (right), co-expressed with hTAS1R2, stimulated with sucralose in the absence (black and green full curves) or presence of 10 mM GSH (black and green dotted curves) (A), and dose-response curves of mutated receptors, co-expressed with hTAS1R1, stimulated with L-Glu in the absence (black and blue full curves) or presence of 1 mM IMP (black and purple dotted curves) (B), GSH (black and green full curves) (C), L-Glu in the absence (black and orange full curves) or presence of 10 mM GSH (black and red dotted curves) (D), and S807 (black and pink full curves) (E). The data are presented as the mean ± sem of 8 wells from 4 independent experiments. * *p* < 0.05, ** *p* < 0.01, *** *p* < 0.001, calculated via ANOVA followed by Dunnett's test (with reference to hTAS1R2-WT/rTAS1R3-WT (A) and hTAS1R1-WT/rTAS1R3-WT (B-E). The *p*-values are presented in Table S1. WT: wild-type; L-Glu: L-glutamic acid; IMP: inosine 5′-monophosphate; GSH: reduced L-glutathione; S807: N-(heptan-4-yl)benzo[d][1,3]dioxole-5-carboxamide; nd: not determined. (For interpretation of the references to colour in this figure legend, the reader is referred to the web version of this article.)

First, through co-expression with hTAS1R2, we tested the ability of GSH to increase the activity of the three mutated receptors stimulated with sucralose. As shown in Fig. 4A, all three mutants responded well to sucralose. The receptor response was not increased by 10 mM GSH for Mut1 and Mut2, whereas the synergy was unaffected in the case of Mut3. These results confirm that GSH increases the sucralose response through binding to the TAS1R3 subunit.

When co-expressed with hTAS1R1, the mutants presented completely different behaviors. As shown in Fig. 4B and D, hTAS1R1/rTAS1R3(Mut1) responded to L-Glu, and this response was increased by the addition of IMP or GSH. In contrast, the response to GSH was reduced, and the response to S807,  which binds to the TMD of TAS1R1, was unaffected (Fig. 4C and E, respectively).

The response of hTAS1R1/rTAS1R3(Mut2) and hTAS1R1/rTAS1R3(Mut3) to L-Glu and GSH was abolished, whereas the response to S807 was severely impaired (Fig. 4D and E). Furthermore, the residual response to S807 in Mut2 and Mut3 receptors, despite the abolition of responses to L-Glu and GSH, suggested that these mutations introduced into the VFT domain of TAS1R3 may affect the function of the VFT of TAS1R1, either through disrupted allosteric communication or through structural alterations such as misfolding or destabilization of the heterodimeric complex. The fact that S807 still elicited a reduced response implied that the overall architecture of the receptor was at least partially preserved, allowing some degree of activation, albeit significantly degraded. Taken together, these results highlight the functional interdependence of the two VFTs.

### 3.3 GSH and TAS1R1 interactions

#### 3.3.1 GSH binds TAS1R1

**Fig. 5:**
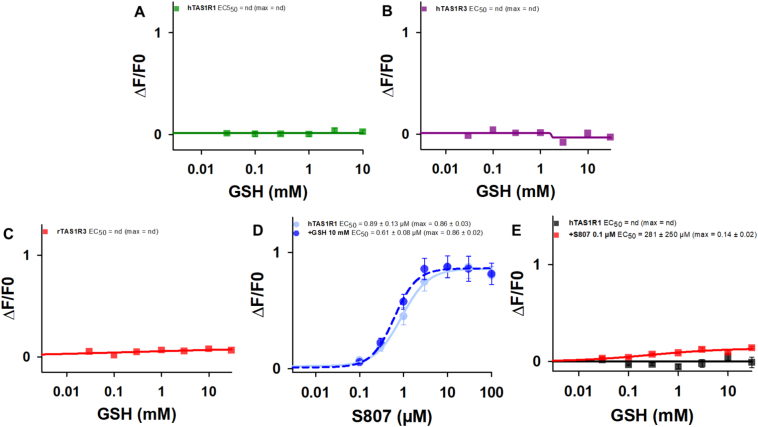
Dose-response curves of hTAS1R1 (A), hTAS1R3 (B), and rTAS1R3 (C) alone stimulated with GSH, and of hTAS1R1 alone stimulated with S807 in the absence or presence of 10 mM GSH (D) and stimulated with GSH in the absence or presence of 0.1 μM S807. The data are presented as the mean ± sem of 8 wells from 4 independent experiments. GSH: reduced L-glutathione; S807: N-(heptan-4-yl)benzo[d][1,3]dioxole-5-carboxamide; nd: not determined.

Cellular assays have shown that in addition to activating the heterodimeric sweet taste receptor (Belloir et al., 2021; Zhao et al., 2018), the sweetener perillartine can activate hTAS1R2 in the absence of hTAS1R3. It has already been reported that hTAS1R3 alone is activated by calcium (Tordoff et al., 2012). We therefore tested the ability of the different TAS1R subunits expressed alone to respond to GSH. We found that HEK293 cells transfected with hTAS1R1, hTAS1R3 or rTAS1R3 did not respond to GSH (Fig. 5A-C). In contrast, S807 induced a dose-dependent response from the hTAS1R1 subunit expressed alone, leading to an EC_50_ value of 0.89 ± 0.13 μM (Fig. 5D), demonstrating that hTAS1R1 in the absence of the TAS1R3 subunit was functional. This response to S807 was not increased in the presence of 10 mM GSH. We then investigated whether S807 could increase the activity of hTAS1R1 stimulated with GSH. Interestingly, the presence of 0.1 μM S807 enabled the response of GSH, leading to an EC_50_ value of 281 ± 250 μM (Fig. 5E). These data demonstrated that GSH bound to the hTAS1R1 subunit.

#### 3.3.2 L-Glu synergizes with GSH

**Fig. 6:**
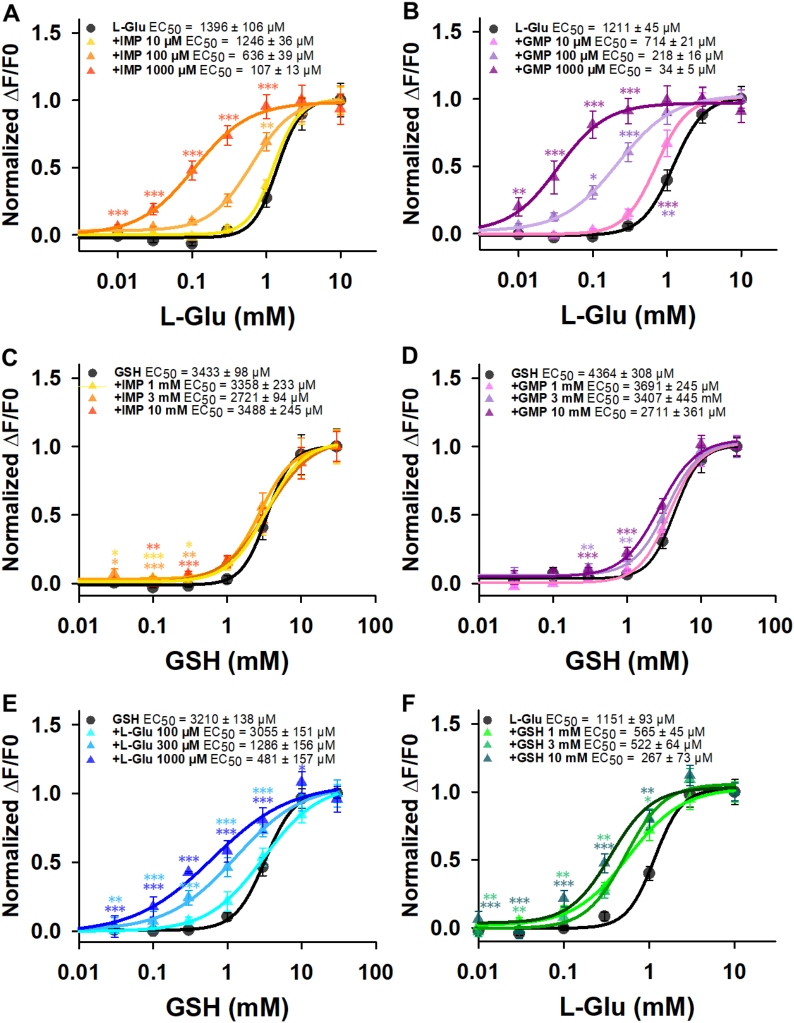
hTAS1R1/rTAS1R3 dose-response curves of L-Glu + IMP (0,10, 100 and 1000 μM) (black and yellow to orange gradient curves) (A), L-Glu + GMP (0, 10, 100 and 1000 μM) (black and pink to purple gradient curves) (B), GSH + IMP (0, 1, 3 and 10 mM) (black and yellow to orange gradient curves) (C), GSH + GMP (0, 1, 3 and 10 mM) (black and pink to purple gradient curves) (D), GSH + L-Glu (0, 100, 300 and 1000 μM) (black and blue gradient curves) (E), and L-Glu + GSH (0, 1, 3 and 10 mM) (black and green gradient curves) (F) The data are presented as the mean ± sem of 8 wells from 4 independent experiments. * *p* < 0.05, ** *p* < 0.01, *** *p* < 0.001, calculated via ANOVA followed by Dunnett's test (with reference to L-Glu and GSH alone for A–C and D–F, respectively). The *p*-values are presented in Table S2. L-Glu: L-glutamic acid; IMP: inosine 5′-monophosphate; GMP: guanosine 5′-monophosphate; GSH: reduced L-glutathione. (For interpretation of the references to colour in this figure legend, the reader is referred to the web version of this article.)

The strong potentiation of L-Glu by the purinine ribonucleotides IMP and GMP is a hallmark of umami taste in humans and in many animal species (Belloir et al., 2017; Sato & Akaike, 1965; Yamaguchi, 1991). Cellular assays have revealed that hTAS1R1/hTAS1R3 responds to the umami taste stimulus L-Glu and that this response is enhanced by 5′-ribonucleotides (Belloir et al., 2017; Li et al., 2002; Raliou et al., 2011; Shigemura et al., 2009). Our data confirmed these synergistic effects (Fig. 6A–B). We investigated whether the hTAS1R1/rTAS1R3 response to GSH could be modulated by IMP and GMP. As expected, the presence of IMP and GMP did not significantly increase the hTAS1R1/rTAS1R3 response to GSH (Fig. 6C–D). This observation was consistent with previous data showing that 1 mM IMP had no effect on the GSH response of TAS1R1/TAS1R3 (Servant & Frerot, 2022). This absence of synergy suggested that GSH and IMP share binding sites localized in hTAS1R1-VFT. We then tested the capacity of L-Glu to potentiate the hTAS1R1/rTAS1R3 response to GSH. Interestingly, we observed that the addition of 300 μM or 1000 μM L-Glu increased the GSH response of hTAS1R1/rTAS1R3 in a dose-dependent manner, leading to 2.5- and 6.7-fold decreases in the EC_50_ value, respectively (Fig. 6E). Reciprocally, in the presence of 3 mM and 10 mM GSH, the dose-response curves of hTAS1R1/rTAS1R3 to L-Glu revealed a leftward shift and a decrease in the EC_50_ values (Fig. 6F).

#### 3.3.3 Mapping the ligand-binding site of GSH onto TAS1R1-VFT

**Fig. 7:**
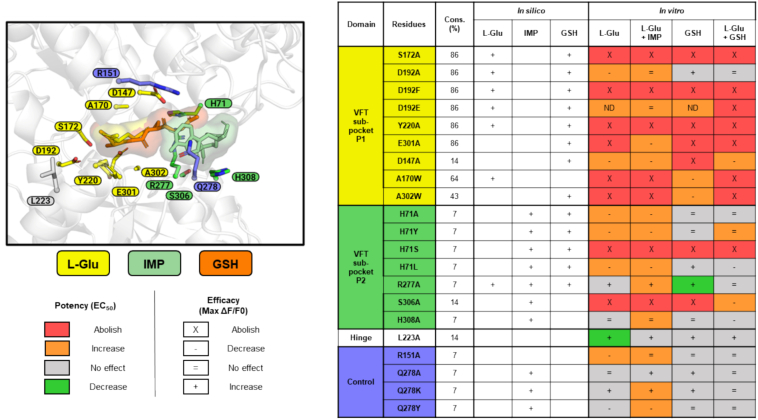
Functional and structural characterization of the GSH binding site in hTAS1R1. Site-directed mutagenesis results evaluating the effects of single amino acid substitutions in hTAS1R1 on ligand interactions (in silico results) and on potency (EC_50_) and efficacy (ΔF/F0) (in vitro results). L-Glu: L-glutamic acid; IMP: inosine 5′-monophosphate; GSH: reduced L-glutathione; VFT: Venus flytrap; Cons.: conservation; ND: not determined.

To identify the critical residues involved in the binding site of GSH, we performed mutagenesis on hTAS1R1-VFT using a homology molecular model as a guide and previously published data (Toda et al., 2013; Zhang et al., 2008). Docked L-Glu aligned with the experimentally observed X-ray pose, occupying a deep binding cavity defined as sub-pocket P1. This region lies near the hinge of the VFT, a crucial structure for ligand binding in class C GPCRs. Mutating the amino acids that interact with L-Glu (S172, D192, Y220, E301, D147, A170, A302) abolished receptor activity (Fig. 7). Among these seven residues, five are highly conserved across class C receptors, suggesting a shared mechanism for L-Glu detection within the VFT sub-pocket P1. Moreover, previous studies on TAS1R1 have shown that mutation of S172, D192, Y220, E301, A302, or A170 affects L-Glu recognition (Toda et al., 2013; Zhang et al., 2008). GSH was also predicted to bind to VFT sub-pocket P1, and its glutamate moiety overlapped precisely with docked glutamate. This structural overlap likely explains why GSH acted as an agonist: VFT sub-pocket P1 recognized the glutamate-like portion of GSH, triggering a response similar to that of L-Glu. These findings highlight a highly conserved binding recognition mechanism in class C GPCRs, where the hinge region and adjacent amino acids in sub-pocket P1 are key to sensing L-Glu and related molecules. In addition to the glutamate moiety, GSH contains an additional cysteinyl glycine group that extends into a neighboring binding region, defined here as VFT sub-pocket P2. This structural analysis led to the identification of twenty-one residues located in the ligand-binding pocket of TAS1R1 as candidates for interaction with L-Glu, IMP, and GSH.

The hTAS1R1 mutants were co-expressed with the rTAS1R3 subunit in HEK293 cells. Cellular responses were assessed after stimulation with L-Glu or GSH alone. We also investigated the ability of IMP and GSH to increase the activity of these mutated receptors stimulated with L-Glu. The dose-response curves for each mutant are presented in **Fig. S4** and the dose-response curves for L-Glu and L-Glu + 1 mM IMP; GSH; and L-Glu and L-Glu + 1 mM GSH are presented in **Fig. S5-S7**, respectively. The EC_50_ values and the maximal signal amplitudes are summarized in Table 1. As a control, the functionality of the mutated receptors was checked after stimulation with S807 (**Fig. S8**), which interacts with TAS1R1-TMD (Zhang et al., 2008). As shown in **Fig. S8**, we found that all the mutants were functional and responded well to S807, with the exception of the S172A, Y220A, D147A, A302W, H71A, R151A, S306A, H308A, and Q278Y mutants, for which the amplitude of the response was strongly affected. These mutations may alter receptor functionality; therefore, the results related to these mutations should be interpreted with caution. The S172A, D192F, Y220A, E301A, A170W, A302W, H71S, and S306A mutations completely suppressed the response to L-Glu. These results partially aligned with those reported by Zhang et al. (2008), who demonstrated that the S172, D192, Y220, and E301 residues were critical for L-Glu activity. However, we observed that the response of D192 was dependent on the volume of the amino acid side chain rather than its charge. Mutating L-Asp to L-Glu (which differs by a single additional methyl group) decreased the receptor response to L-Glu. Similarly, the introduction of a bulkier side chain, such as *L*-phenylalanine at position D192, abolished the receptor response to L-Glu stimulation. In contrast, replacement by a small nonpolar residue, such as L-alanine, preserved agonist-induced receptor activation. We found that the synergy between L-Glu and IMP was suppressed for the H71A, H71Y, H71L, R277A, and H308A mutants (**Fig. S5**). The different mutations of the H71 residue demonstrated that this residue was essential for the interaction of hTAS1R1-VFT with IMP. Interestingly, a functional response was observed in D192E and E301A mutants when IMP and L-Glu were applied together rather than L-Glu alone. Our results were partially consistent with those of a previous study showing that the H71, R277, S306, and H308 residues are involved in IMP activity (Zhang et al., 2008). Our results provide further details on the key residues involved in L-Glu and IMP binding to TAS1R1-VFT.

**Table 1:** EC_50_ values (μM) and maximal amplitudes (max ∆F/F0) measured for TAS1R1-WT or TAS1R1 mutants co-expressed with TAS1R3 and stimulated with L-Glu alone, L-Glu + 1 mM IMP, GSH, L-Glu alone and L-Glu + 1 mM GSH.

Domain	Mutants	L-Glu (IMP series)				L-Glu + 1 mM IMP				GSH				L-Glu (GSH series)				L-Glu + 1 mM GSH			
		EC_50_	R1	Max ∆F/F0	R2	Ec_50_	R1	Max ∆F/F0	R2	EC_50_	R1	Max ∆F/F0	R2	EC_50_	R1	Max ∆F/F0	R2	EC_50_	R1	Max ∆F/F0	R2
–	WT	1815 ± 87	1.0	0.90 ± 0.01	1.0	113 ± 20	1.0	1.04 ± 0.03	1.0	2522 ± 238	1.0	0.23 ± 0.01	1.0	1402 ± 24	1.0	1.62 ± 0.01	1.0	631 ± 93	1.0	1.48 ± 0.07	1.0
VFT sub-pocket P1	S172A	–	–	–	–	–	–	–	–	–	–	–	–	–	–	–	–	–	–	–	–
	D192A	19,214 ± 385	10.6	0.84 ± 0.01	0.9	1397 ± 159	12.4	1.04 ± 0.02	1.0	3246 ± 61	1.3	0.65 ± 0.01	2.8	1376 ± 46	1.0	1.92 ± 0.03	1.2	648 ± 61	1.0	1.85 ± 0.06	1.2
	D192F	–	–	–	–	–	–	–	–	–	–	–	–	–	–	–	–	–	–	–	–
	D192E	> 100,000	–	–	–	5429 ± 1027	48.0	0.96 ± 0.06	0.9	–	–	–	–	> 30,000	–	–	–	–	–	–	–
	Y220A	–	–	–	–	–	–	–	–	–	–	–	–	–	–	–	–	–	–	–	–
	E301A	–	–	–	–	5215 ± 225	46.1	0.74 ± 0.02	0.7	–	–	–	–	–	–	–	–	–	–	–	–
	D147A	3512 ± 190	1.9	0.17 ± 0.01	0.2	603 ± 228	5.3	0.35 ± 0.03	0.3	–	–	–	–	4264 ± 1409	3.0	0.60 ± 0.11	0.4	2434 ± 1401	3.9	0.60 ± 0.10	0.4
	A170W	–	–	–	–	–	–	–	–	5293 ± 1565	2.1	0.09 ± 0.01	0.3	–	–	–	–	–	–	–	–
	A302W	–	–	–	–	–	–	–	–	5316 ± 2540	2.1	0.09 ± 0.01	0.3	–	–	–	–	–	–	–	–
VFT sub-pocket P2	H71A	8686 ± 2424	4.8	0.44 ± 0.04	0.5	8501 ± 1444	75.2	0.41 ± 0.02	0.4	2030 ± 416	0.8	0.44 ± 0.03	1.9	1614 ± 271	1.1	1.13 ± 0.08	0.7	1011 ± 849	1.6	0.96 ± 0.26	0.7
	H71Y	6000 ± 249	3.3	0.63 ± 0.09	0.7	3813 ± 136	33.7	0.65 ± 0.01	0.6	12,391 ± 3986	4.9	0.65 ± 0.11	2.8	4829 ± 2128	3.4	1.33 ± 0.36	0.8	4065 ± 785	6.4	1.38 ± 0.14	0.9
	H71S	–	–	–	–	–	–	–	–	–	–	–	–	–	–	–	–	–	–	–	–
	H71L	3303 ± 389	1.8	0.58 ± 0.03	0.6	2260 ± 599	20.0	0.55 ± 0.05	0.5	2268 ± 123	0.9	1.24 ± 0.0	5.4	2434 ± 30	1.7	1.32 ± 0.01	0.8	1432 ± 959	2.3	0.26 ± 0.09	0.2
	R277A	2704 ± 305	1.5	1.62 ± 0.06	1.8	2014 ± 417	17.8	1.56 ± 0.10	1.5	1842 ± 174	0.7	0.85 ± 0.02	3.7	1856 ± 62	1.3	1.28 ± 0.02	0.8	1278 ± 147	2.0	1.21 ± 0.06	0.8
	S306A	–	–	–	–	–	–	–	–	–	–	–	–	3088 ± 418	2.2	0.20 ± 0.02	0.1	1325 ± 232	2.1	0.22 ± 0.02	0.2
	H308A	1429 ± 277	0.8	0.98 ± 0.07	1.1	1119 ± 98	9.8	1.11 ± 0.03	0.1	3251 ± 225	1.3	0.42 ± 0.01	1.9	1219 ± 142	0.9	0.96 ± 0.05	0.6	518 ± 110	0.8	1.13 ± 0.07	0.8
Hinge	L223A	1098 ± 245	0.6	1.43 ± 0.11	1.6	61 ± 77	0.5	1.68 ± 0.16	1.6	2317 ± 308	0.9	1.71 ± 0.09	7.4	681 ± 72	0.5	2.08 ± 0.08	1.3	318 ± 25	0.5	1.67 ± 0.04	1.1
Control	R151A	3240 ± 1248	1.8	0.43 ± 0.05	0.5	438 ± 126	3.9	0.98 ± 0.06	0.9	2593 ± 1594	1.0	0.31 ± 0.08	1.3	3060 ± 168	2.2	1.39 ± 0.04	0.9	2316 ± 604	3.7	1.62 ± 0.02	1.1
	Q278A	1541 ± 492	0.8	1.09 ± 0.10	1.2	86 ± 48	0.8	1.41 ± 0.14	1.4	3419 ± 73	1.4	1.46 ± 0.01	6.3	1586 ± 48	1.1	1.76 ± 0.03	1.1	637 ± 36	1.0	1.68 ± 0.03	1.1
	Q278K	2817 ± 687	1.6	1.22 ± 0.12	1.4	332 ± 216	2.9	1.60 ± 0.03	1.5	5662 ± 310	2.2	1.10 ± 0.02	4.7	2553 ± 263	1.8	1.95 ± 0.11	1.2	1303 ± 270	2.1	1.88 ± 0.15	1.3
	Q278Y	1889 ± 813	1.0	0.43 ± 0.05	0.5	298 ± 150	2.6	0.62 ± 0.07	0.6	3961 ± 1760	1.6	0.47 ± 0.07	2.0	1680 ± 47	1.2	1.35 ± 0.02	0.8	921 ± 95	1.5	1.40 ± 0.05	0.9

R1 = mutant EC_50_/WT EC_50_; R1 > 1 represents a decrease in agonist potency at the receptor level.

R2 = (mutant Max ΔF/F0)/(WT Max ΔF/F0); R2 < 1 represents a decrease in agonist efficacy at the receptor level.

WT: wild-type; VFT; Venus flytrap domain; L-Glu: L-glutamic acid; IMP: inosine 5′-monophosphate; “-”: no activation.

Finally, the mutagenesis analysis confirmed that GSH bound to hTAS1R1-VFT. Mutations in this area interfere with the hTAS1R1/rTAS1R3 response to GSH. The mutations that fully abolished the response to GSH were S172A, D192F, Y220A, E301A, D147A, H71S, and S306A (**Fig. S6**). For most of these residues, mutation to L-alanine, a small, neutral, and nonpolar amino acid, resulted in no response to GSH. Interestingly, the D192A, H71L, R277A, L223A, Q278A, and Q278K mutations significantly increased the initial amplitude of GSH compared with that of hTAS1R1-WT/rTAS1R3. R277A and L223A mutants also increased the amplitude to L-Glu compared to that in the hTAS1R1-WT/rTAS1R3 (**Fig. S4**). These six positions play a role in agonist interactions with the receptor. In the presence of 1 mM GSH (**Fig. S7**), synergy was observed for the following mutants: D192A, H308A, L223A, Q278A, Q278K, and Q278Y. In contrast the addition of GSH did not affect the dose-response curves for the D147A, H71A, H71Y, R151A, R277A, and S306A mutants, suggesting that they participate in this interaction.

We identified two mutations that completely abolished the response to L-Glu, GSH, L-Glu + IMP, and L-Glu + GSH, namely, D192F and H71S, whereas the response to S807 was increased and unaffected, respectively. These amino acid residues are critically involved in the interaction between these molecules and hTAS1R1. These results confirmed that L-Glu, IMP and GSH interact with the umami taste receptor in the same area located in TAS1R1-VFT and share common key residues.

## 4 Conclusion

Our study provides important insights into the molecular mechanisms by which GSH interacts with taste receptors, underscoring its dual significance for food-flavor science. We demonstrated that GSH acts as a partial agonist of hTAS1R1/rTAS1R3, with lower efficacy than L-Glu. Through a combination of cellular assays, molecular modeling, and site-directed mutagenesis, we mapped the binding site of GSH to hTAS1R1-VFT, which overlaps with the binding sites of L-Glu and IMP. We showed a novel synergistic interaction between GSH and L-Glu that was not observed between GSH and 5′-ribonucleotides (IMP and GMP). This suggested that while GSH and L-Glu may simultaneously bind to the umami taste receptor and potentiate their respective effects, GSH and ribonucleotides likely compete for overlapping binding sites within TAS1R1-VFT. Furthermore, our research revealed that GSH could also activate the sweet taste receptor TAS1R2/TAS1R3 and potentiate its response to sucralose. Through chimeric receptor studies and site-directed mutagenesis, we demonstrated that this interaction occurred for rTAS1R3-VFT but not hTAS1R3-VFT, highlighting important species differences in ligand recognition. The identification of key residues involved in the binding of GSH to both TAS1R1-VFT and TAS1R3-VFT provides a structural basis for understanding how this tripeptide interacts with taste receptors. Our mutagenesis studies identified critical amino acids that mediate GSH binding and receptor activation, as mutations at positions D192F and H71S completely abolished the responses to both L-Glu and GSH.

These findings suggest that the kokumi activity of GSH is more complex than previously thought and extends beyond its known interaction with CaSR. GSH interacts with multiple taste receptors, including TAS1R1/TAS1R3 and TAS1R2/TAS1R3, which may explain its ability to enhance different taste modalities. This research provides a better understanding of the molecular mechanisms underlying taste detection and introduces new opportunities for the development of taste enhancers or modulators, particularly in the formulation of food products, that target specific taste receptors. Future studies should explore the physiological relevance of these interactions in vivo and investigate whether other kokumi compounds share similar mechanisms of action.

## CRediT authorship contribution statement

**Clémence Cornut:** Writing – review & editing, Writing – original draft, Visualization, Investigation, Formal analysis, Conceptualization. **Adeline Karolkowski:** Writing – review & editing, Writing – original draft, Visualization, Formal analysis. **Maxence Lalis:** Writing – review & editing, Writing – original draft, Visualization, Investigation, Formal analysis. **Antoine Thomas:** Project administration. **Rudy Menin:** Project administration. **Jérémie Topin:** Writing – review & editing, Supervision, Methodology, Conceptualization. **Loïc Briand:** Writing – review & editing, Writing – original draft, Supervision, Project administration, Funding acquisition, Conceptualization. **Christine Belloir:** Writing – review & editing, Writing – original draft, Visualization, Supervision, Resources, Project administration, Methodology, Investigation, Funding acquisition, Formal analysis, Conceptualization.

## Funding

The PhD of Clémence Cornut was funded by Biospringer by Lesaffre (France) and 10.13039/501100003032ANRT (National Agency for Research and Technology, France) (ANRT-CIFRE 2022/0563). They did not have any influence over the study conception, design, interpretation, or the decision to publish the data.

## Declaration of competing interest

The authors declare the following financial interests/personal relationships which may be considered as potential competing interests: Loic BRIAND reports financial support was provided by Biospringer by Lesaffre. Clemence CORNUT reports financial support was provided by Biospringer by Lesaffre. Loic BRIAND reports a relationship with Biospringer by Lesaffre that includes: funding grants. If there are other authors, they declare that they have no known competing financial interests or personal relationships that could have appeared to influence the work reported in this paper.

## Data Availability

Data will be made available on request.
