# Supplementary material for: Glutathione as a taste modulator: molecular mechanisms of interaction with umami and sweet taste receptors
Source: Food Chem (Oxf). 2025 Oct 26;11:100319. doi: 10.1016/j.fochms.2025.100319 (PMC12615753; doi:10.1016/j.fochms.2025.100319)
Supplement: Supplementary material [file mmc1.docx]

**Glutathione as a taste modulator: molecular mechanisms of interaction with umami and sweet taste receptors**

Clémence Cornut, Adeline Karolkowski, Maxence Lalis, Antoine Thomas, Rudy Menin, Jérémie Topin, Loïc Briand, Christine Belloir

# Supplementary data

- **Protein sequence of the chimeric rat Gα15i2**

The last five residues of the C-terminal tail of rat Gα15i2 (EINLL) (UniProt number G3V6N8) were replaced by the counterpart sequence from human GNAI2 (DCGLF) (UniProt number P04899-1), shown in bold in the sequence below.

MARSLTWGCCPWCLTEEEKTAARIDQEINKILLEQKKQERGELKLLLLGPGESGKSTFIKQMRIIHGAGYSEEDRRAFRLLVYQNIFVSMQAMIEAMDRLQIPFSRPDSKQHASLVMTQDPYKVSSFEKPYAVAMQYLWRDAGIRACYERRREFHLLDSAVYYLSHLERIAEDDYIPTAQDVLRSRMPTTGINEYCFSVQKTKLRIVDVGGQKSERKKWIHCFENVIALIYLASLSEYDQCLEENSQENRMKESLALFSTILELPWFKSTSVILFLNKTDILEDKIHTSHLASYFPSFQGPRRDAEAAKRFILDMYARVYASCAEPHDGGRKGSRARRLFAHFTCATDTHSVRSVFKDVRDSVLARYLD**DCGLF**

- **Protein sequence of human TAS1R1-WT (wild-type) (UniProtKB number Q7RTX1-1)**

MLLCTARLVGLQLLISCCWAFACHSTESSPDFTLPGDYLLAGLFPLHSGCLQVRHRPEVTLCDRSCSFNEHGYHLFQAMRLGVEEINNSTALLPNITLGYQLYDVCSDSANVYATLRVLSLPGQHHIELQGDLLHYSPTVLAVIGPDSTNRAATTAALLSPFLVPMISYAASSETLSVKRQYPSFLRTIPNDKYQVETMVLLLQKFGWTWISLVGSSDDYGQLGVQALENQATGQGICIAFKDIMPFSAQVGDERMQCLMRHLAQAGATVVVVFSSRQLARVFFESVVLTNLTGKVWVASEAWALSRHITGVPGIQRIGMVLGVAIQKRAVPGLKAFEEAYARADKKAPRPCHKGSWCSSNQLCRECQAFMAHTMPKLKAFSMSSAYNAYRAVYAVAHGLHQLLGCASGACSRGRVYPWQLLEQIHKVHFLLHKDTVAFNDNRDPLSSYNIIAWDWNGPKWTFTVLGSSTWSPVQLNINETKIQWHGKDNQVPKSVCSSDCLEGHQRVVTGFHHCCFECVPCGAGTFLNKSDLYRCQPCGKEEWAPEGSQTCFPRTVVFLALREHTSWVLLAANTLLLLLLLGTAGLFAWHLDTPVVRSAGGRLCFLMLGSLAAGSGSLYGFFGEPTRPACLLRQALFALGFTIFLSCLTVRSFQLIIIFKFSTKVPTFYHAWVQNHGAGLFVMISSAAQLLICLTWLVVWTPLPAREYQRFPHLVMLECTETNSLGFILAFLYNGLLSISAFACSYLGKDLPENYNEAKCVTFSLLFNFVSWIAFFTTASVYDGKYLPAANMMAGLSSLSSGFGGYFLPKCYVILCRPDLNSTEHFQASIQDYTRRCGST

- **Protein sequence of rat TAS1R3 (UniProtKB number Q923K1)**

MPGLAILGLSLAAFLELGMGSSLCLSQQFKAQGDYILGGLFPLGTTEEATLNQRTQPNGILCTRFSPLGLFLAMAMKMAVEEINNGSALLPGLRLGYDLFDTCSEPVVTMKPSLMFMAKVGSQSIAAYCNYTQYQPRVLAVIGPHSSELALITGKFFSFFLMPQVSYSASMDRLSDRETFPSFFRTVPSDRVQLQAVVTLLQNFSWNWVAALGSDDDYGREGLSIFSGLANSRGICIAHEGLVPQHDTSGQQLGKVVDVLRQVNQSKVQVVVLFASARAVYSLFSYSILHDLSPKVWVASESWLTSDLVMTLPNIARVGTVLGFLQRGALLPEFSHYVETRLALAADPTFCASLKAELDLEERVMGPRCSQCDYIMLQNLSSGLMQNLSAGQLHHQIFATYAAVYSVAQALHNTLQCNVSHCHTSEPVQPWQLLENMYNMSFRARDLTLQFDAKGSVDMEYDLKMWVWQSPTPVLHTVGTFNGTLQLQHSKMYWPGNQVPVSQCSRQCKDGQVRRVKGFHSCCYDCVDCKAGSYRKHPDDFTCTPCGKDQWSPEKSTTCLPRRPKFLAWGEPAVLSLLLLLCLVLGLTLAALGLFVHYWDSPLVQASGGSLFCFGLICLGLFCLSVLLFPGRPRSASCLAQQPMAHLPLTGCLSTLFLQAAEIFVESELPLSWANWLCSYLRGPWAWLVVLLATLVEAALCAWYLMAFPPEVVTDWQVLPTEVLEHCRMRSWVSLGLVHITNAVLAFLCFLGTFLVQSQPGRYNRARGLTFAMLAYFIIWVSFVPLLANVQVAYQPAVQMGAILFCALGILATFHLPKCYVLLWLPELNTQEFFLGRSPKEASDGNSGSSEATRGHSE

- **Protein sequence of chimera hTAS1R3(r1-575)**

The human Venus flytrap domain (VFT) and the cysteine-rich-domain (CRD) (in bold and italics/red) were replaced by their counterpart sequences from rat (in bold).

**MPGLAILGLSLAAFLELGMGSSLCLSQQFKAQGDYILGGLFPLGTTEEATLNQRTQPNGILCTRFSPLGLFLAMAMKMAVEEINNGSALLPGLRLGYDLFDTCSEPVVTMKPSLMFMAKVGSQSIAAYCNYTQYQPRVLAVIGPHSSELALITGKFFSFFLMPQVSYSASMDRLSDRETFPSFFRTVPSDRVQLQAVVTLLQNFSWNWVAALGSDDDYGREGLSIFSGLANSRGICIAHEGLVPQHDTSGQQLGKVVDVLRQVNQSKVQVVVLFASARAVYSLFSYSILHDLSPKVWVASESWLTSDLVMTLPNIARVGTVLGFLQRGALLPEFSHYVETRLALAADPTFCASLKAELDLEERVMGPRCSQCDYIMLQNLSSGLMQNLSAGQLHHQIFATYAAVYSVAQALHNTLQCNVSHCHTSEPVQPWQLLENMYNMSFRARDLTLQFDAKGSVDMEYDLKMWVWQSPTPVLHTVGTFNGTLQLQHSKMYWPGNQVPVSQ*CSRQCKDGQVRRVKGFHSCCYDCVDCKAGSYRKHPDDFTCTPCGKDQWSPEKSTTCLPRRPKFLAWGEPAVL***LLLLLLSLALGLVLAALGLFVHHRDSPLVQASGGPLACFGLVCLGLVCLSVLLFPGQPSPARCLAQQPLSHLPLTGCLSTLFLQAAEIFVESELPLSWADRLSGCLRGPWAWLVVLLAMLVEVALCTWYLVAFPPEVVTDWHMLPTEALVHCRTRSWVSFGLAHATNATLAFLCFLGTFLVRSQPGCYNRARGLTFAMLAYFITWVSFVPLLANVQVVLRPAVQMGALLLCVLGILAAFHLPRCYLLMRQPGLNTPEFFLGGGPGDAQGQNDGNTGNQGKHE

- **Protein sequence of chimera hTAS1R3(r570-858).**

The human transmembrane domain (TMD) was replaced with its counterpart sequence from rat (in bold).

MLGPAVLGLSLWALLHPGTGAPLCLSQQLRMKGDYVLGGLFPLGEAEEAGLRSRTRPSSPVCTRFSSNGLLWALAMKMAVEEINNKSDLLPGLRLGYDLFDTCSEPVVAMKPSLMFLAKAGSRDIAAYCNYTQYQPRVLAVIGPHSSELAMVTGKFFSFFLMPQVSYGASMELLSARETFPSFFRTVPSDRVQLTAAAELLQEFGWNWVAALGSDDEYGRQGLSIFSALAAARGICIAHEGLVPLPRADDSRLGKVQDVLHQVNQSSVQVVLLFASVHAAHALFNYSISSRLSPKVWVASEAWLTSDLVMGLPGMAQMGTVLGFLQRGAQLHEFPQYVKTHLALATDPAFCSALGEREQGLEEDVVGQRCPQCDCITLQNVSAGLNHHQTFSVYAAVYSVAQALHNTLQCNASGCPAQDPVKPWQLLENMYNLTFHVGGLPLRFDSSGNVDMEYDLKLWVWQGSVPRLHDVGRFNGSLRTERLKIRWHTSDNQKPVSR***CSRQCQEGQVRRVKGFHSCCYDCVDCEAGSYRQNPDDIACTFCGQDEWSPERSTRCFRRRSRFLAWGEPAVL*SLLLLLCLVLGLTLAALGLFVHYWDSPLVQASGGSLFCFGLICLGLFCLSVLLFPGRPRSASCLAQQPMAHLPLTGCLSTLFLQAAEIFVESELPLSWANWLCSYLRGPWAWLVVLLATLVEAALCAWYLMAFPPEVVTDWQVLPTEVLEHCRMRSWVSLGLVHITNAVLAFLCFLGTFLVQSQPGRYNRARGLTFAMLAYFIIWVSFVPLLANVQVAYQPAVQMGAILFCALGILATFHLPKCYVLLWLPELNTQEFFLGRSPKEASDGNSGSSEATRGHSE**


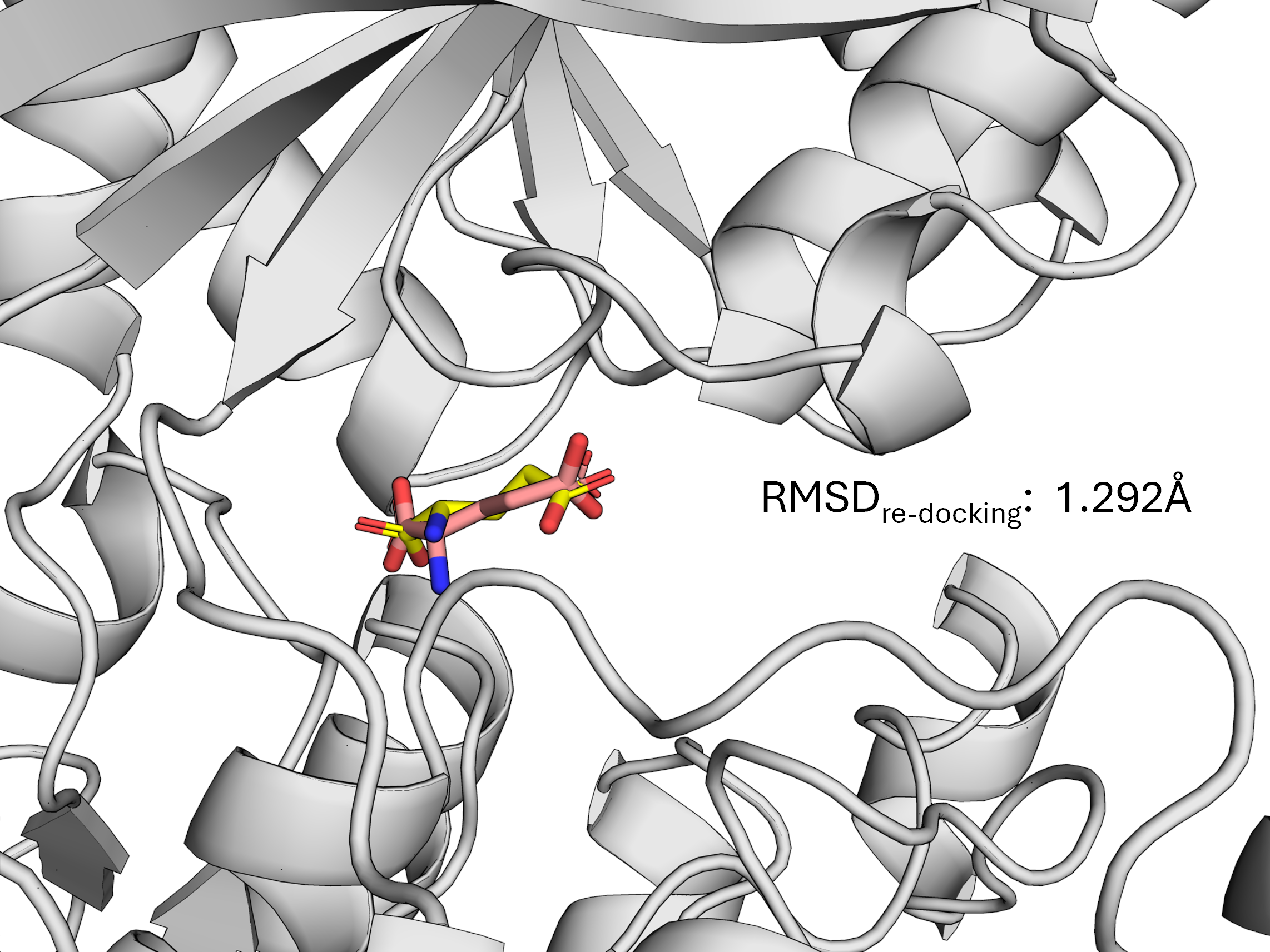


**Figure S1.** Comparison between the crystallographic pose of the L-Glu inside the mGluR1-VFT (in yellow) and the first ranked docked pose (in pink). L-Glu: L-glutamic acid; VFT: Venus flytrap domain.


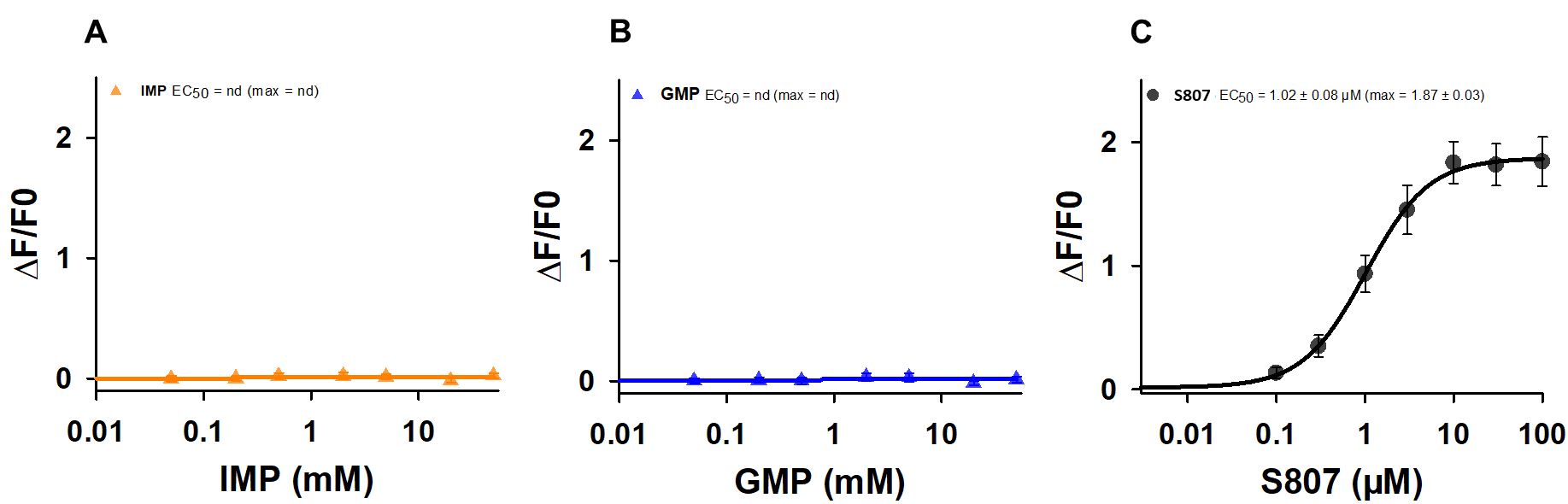


**Figure S2.** Dose-response curves of hTAS1R1/rTAS1R3 stimulated with IMP (A), GMP (B), and S807 (C). The data are presented as the mean ± sem of 8 wells from 4 independent experiments. IMP: inosine 5’-monophosphate; GMP: guanosine 5’-monophosphate; S807: N-(heptan-4-yl)benzo[d][1,3]dioxole-5-carboxamide; nd: not determined.


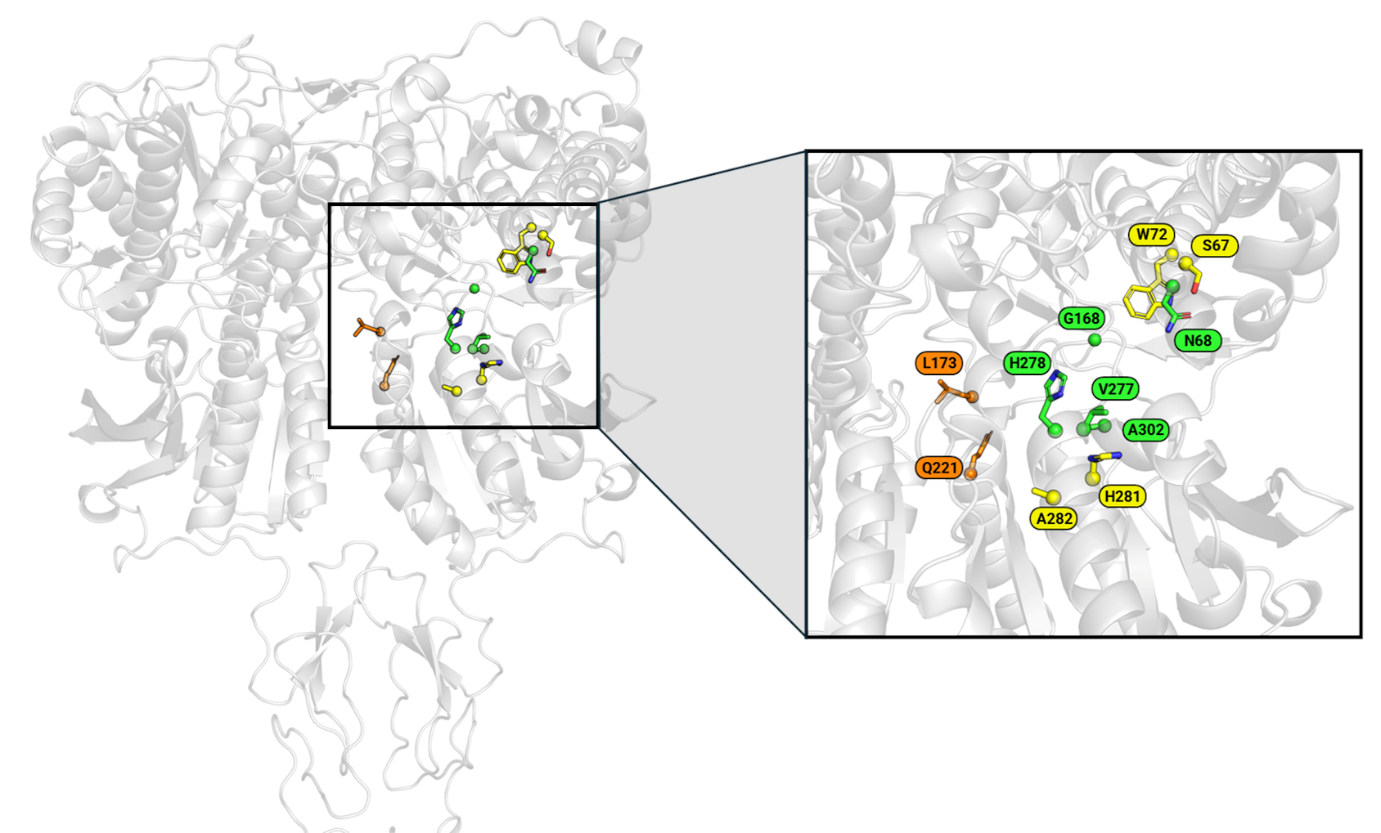


**Figure S3.** Putative binding site of GSH (reduced L-glutathione) in hTAS1R3. The overall architecture of the hTAS1R2/hTAS1R3-VFT heterodimer is shown in cartoon representation in gray. Key amino acid residues putatively involved in ligand interactions are highlighted. Residues from hTAS1R3 are labeled and color-coded according to the directed mutagenesis strategy: green for TAS1R3(Mut1), located deep in the binding cavity (N68, G168, H278, V277, A302); yellow for TAS1R3(Mut2), corresponding to residues surrounding the cavity which differ from species to species and are not directly involved in ligand binding (S67, W72, H281, A282); and orange for TAS1R3(Mut3), which corresponds to residues within the heterodimer interface (L173, Q221).


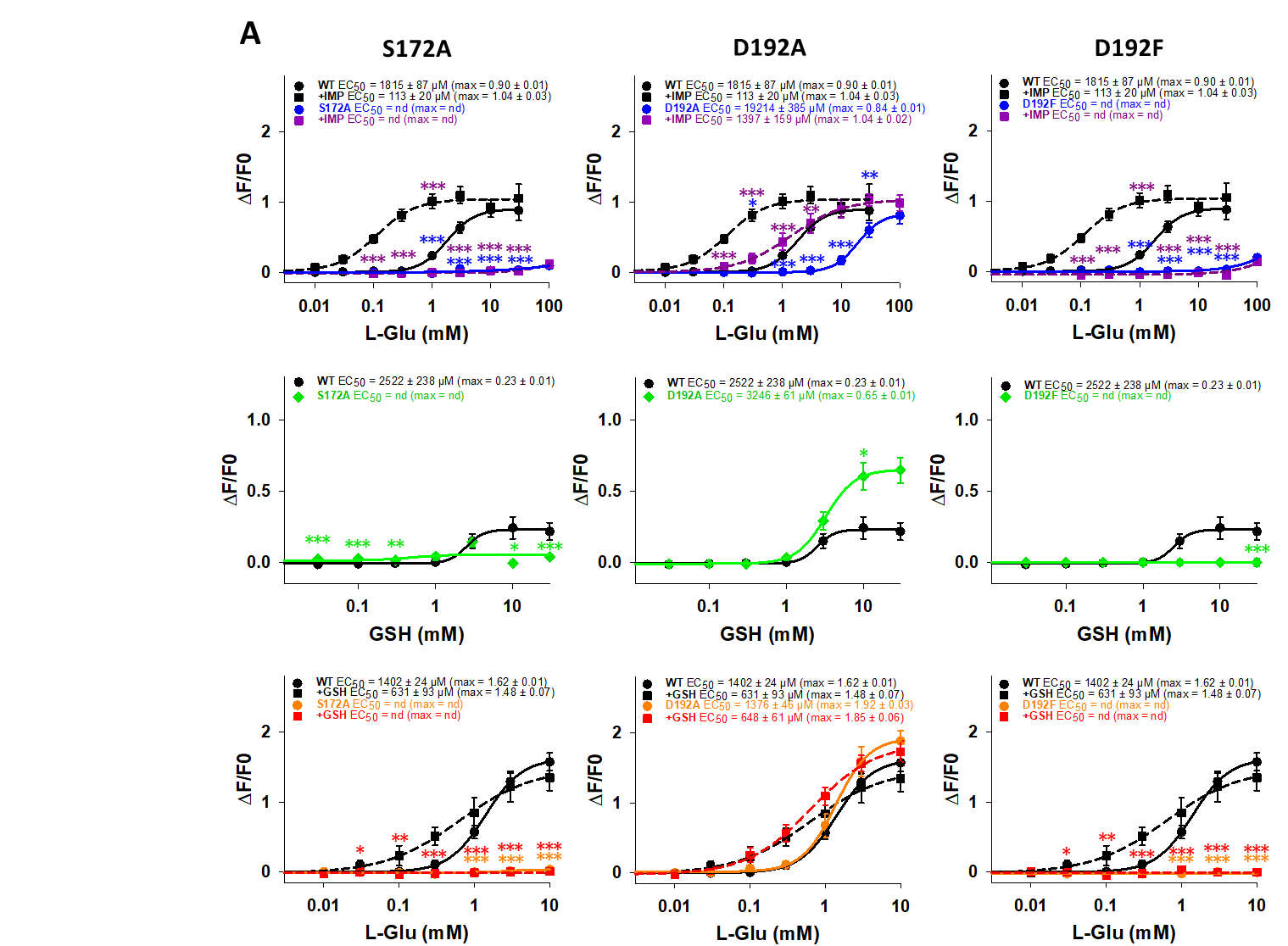

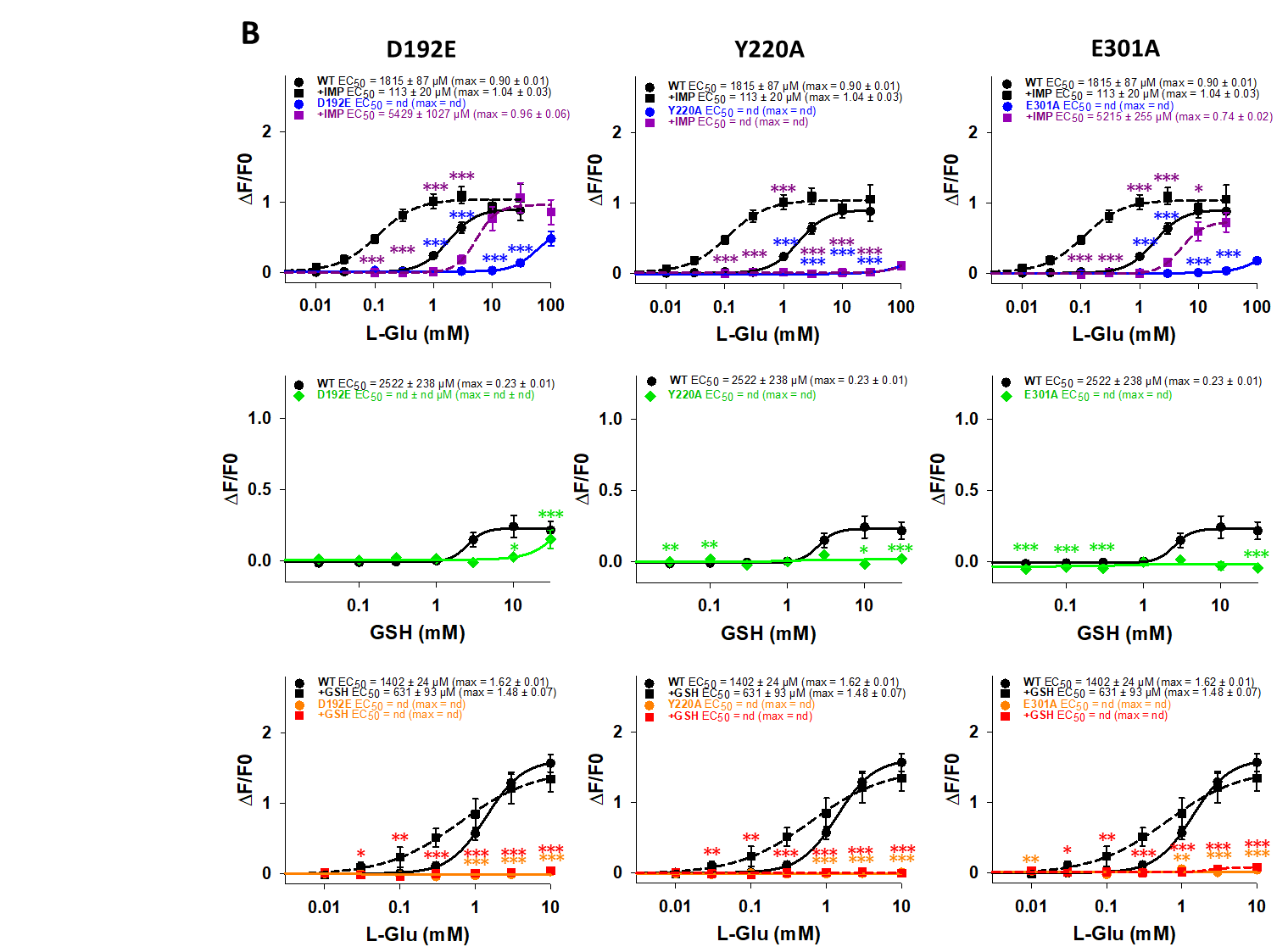

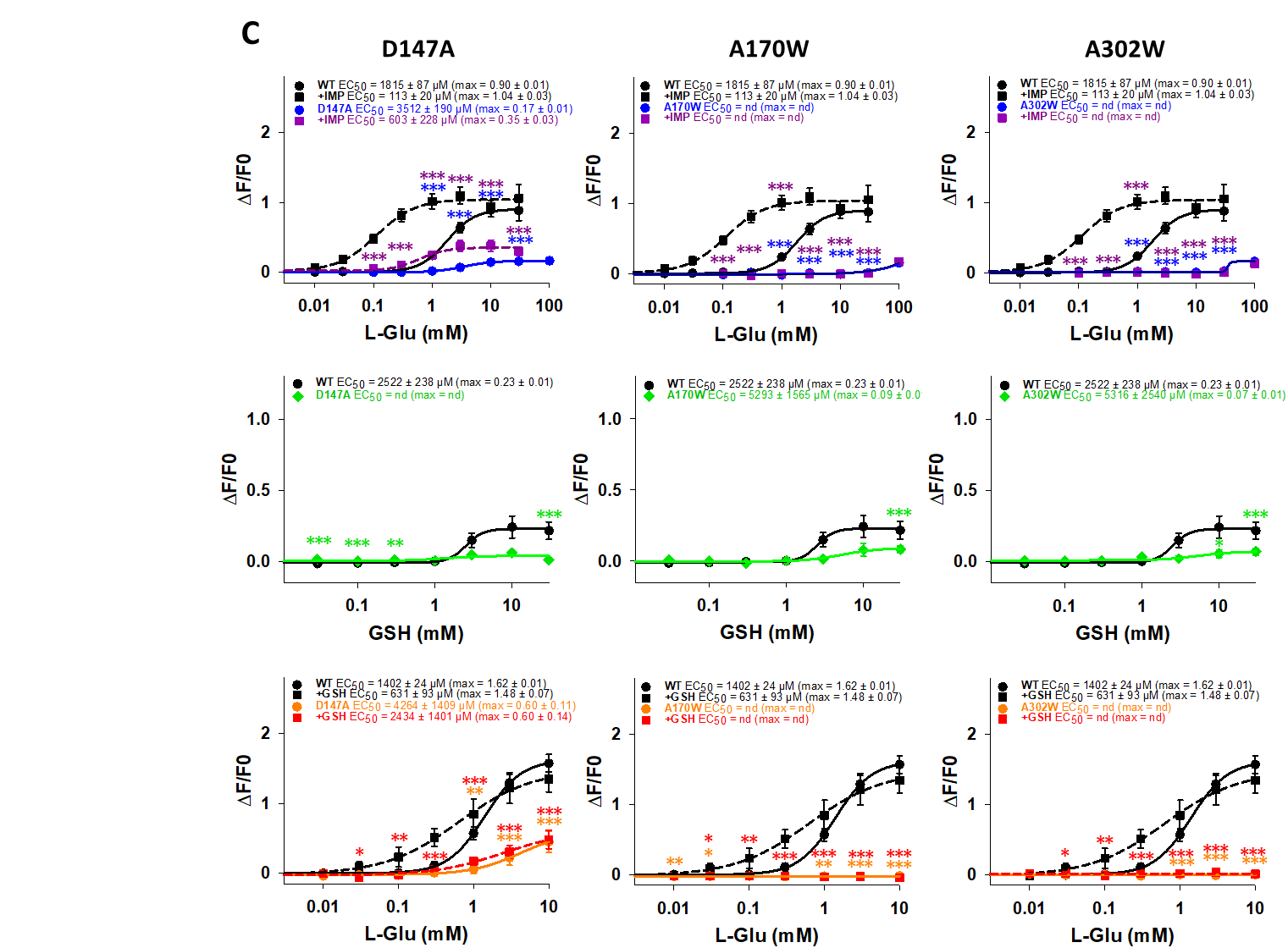

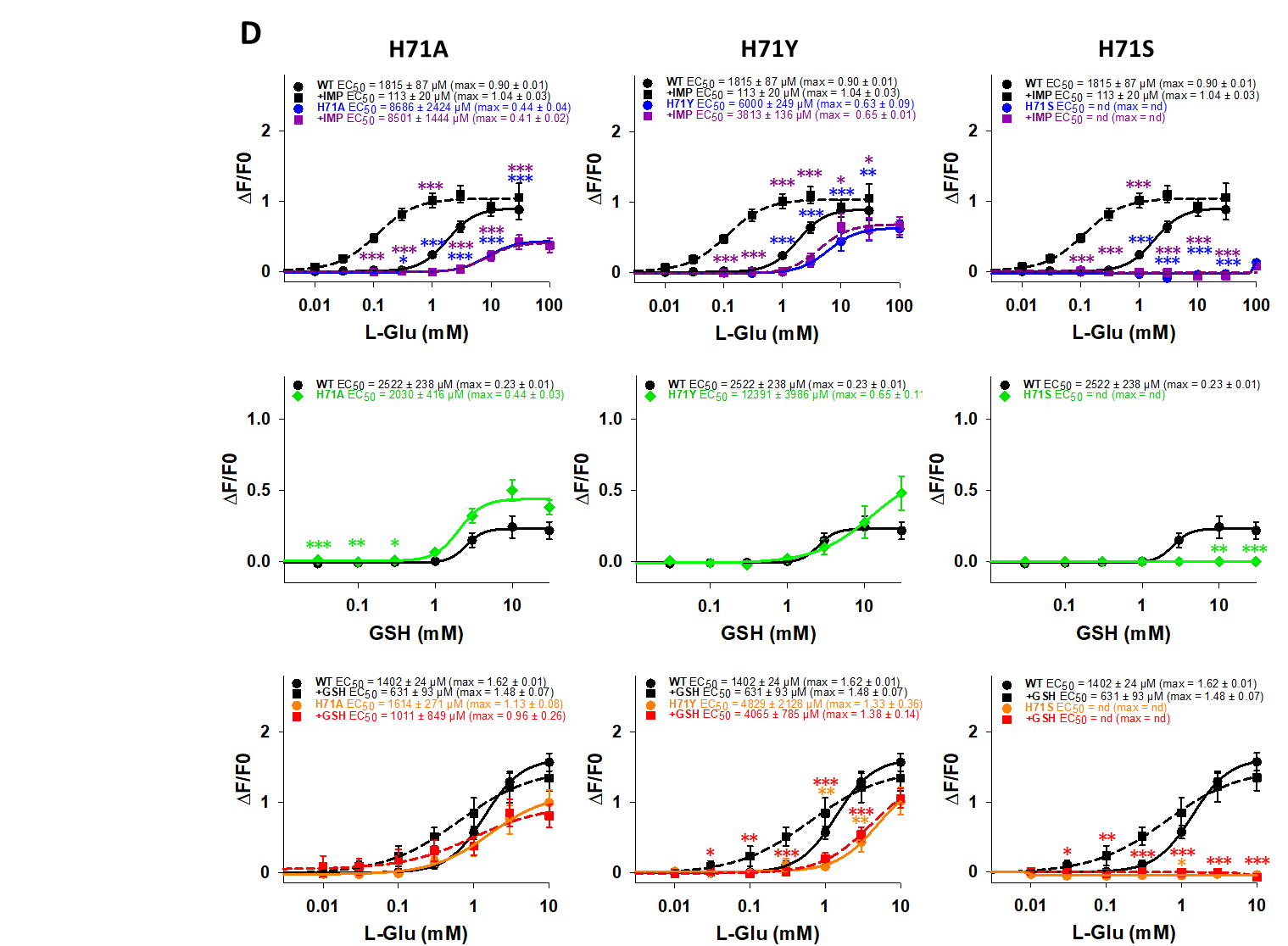

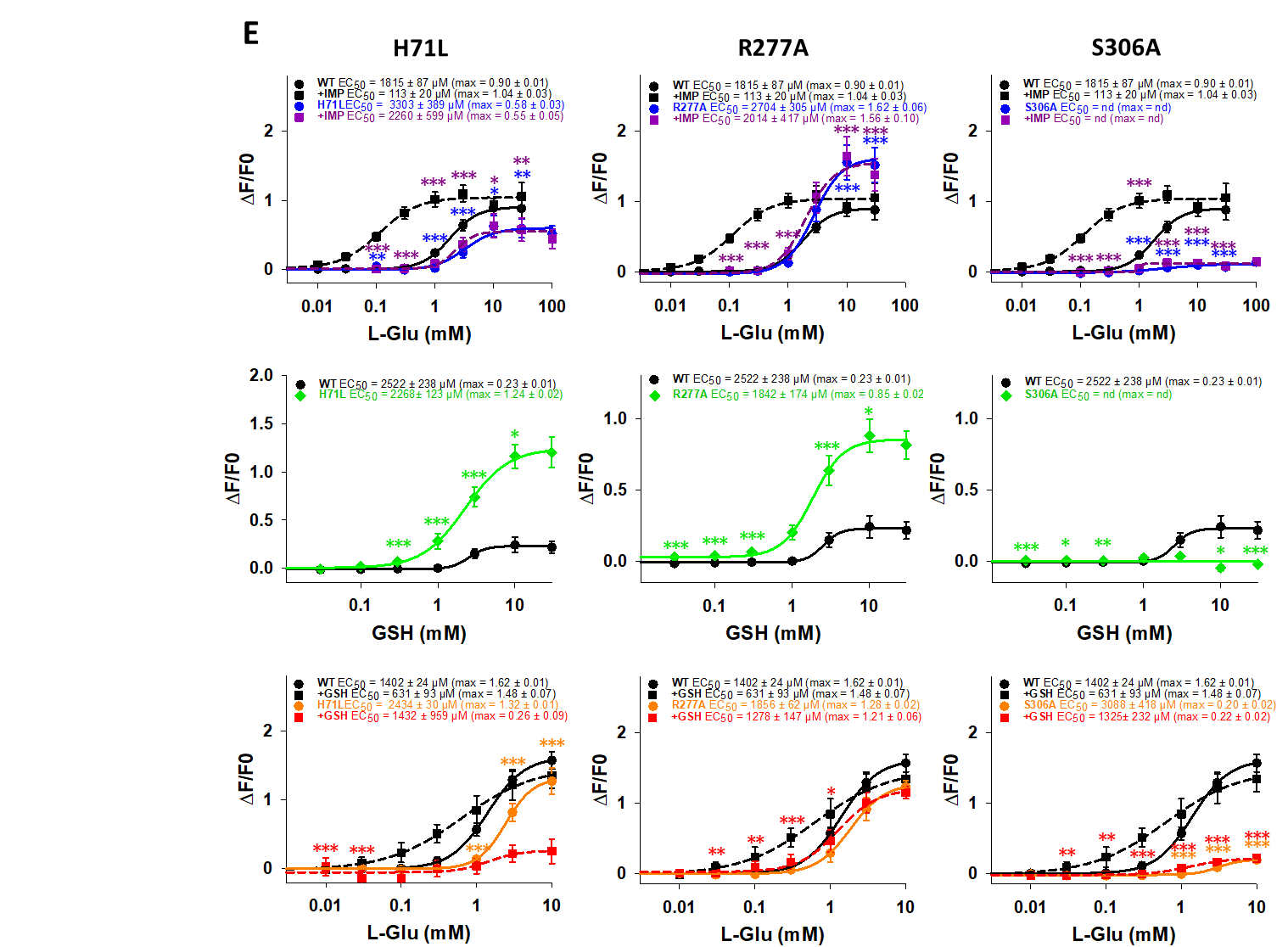

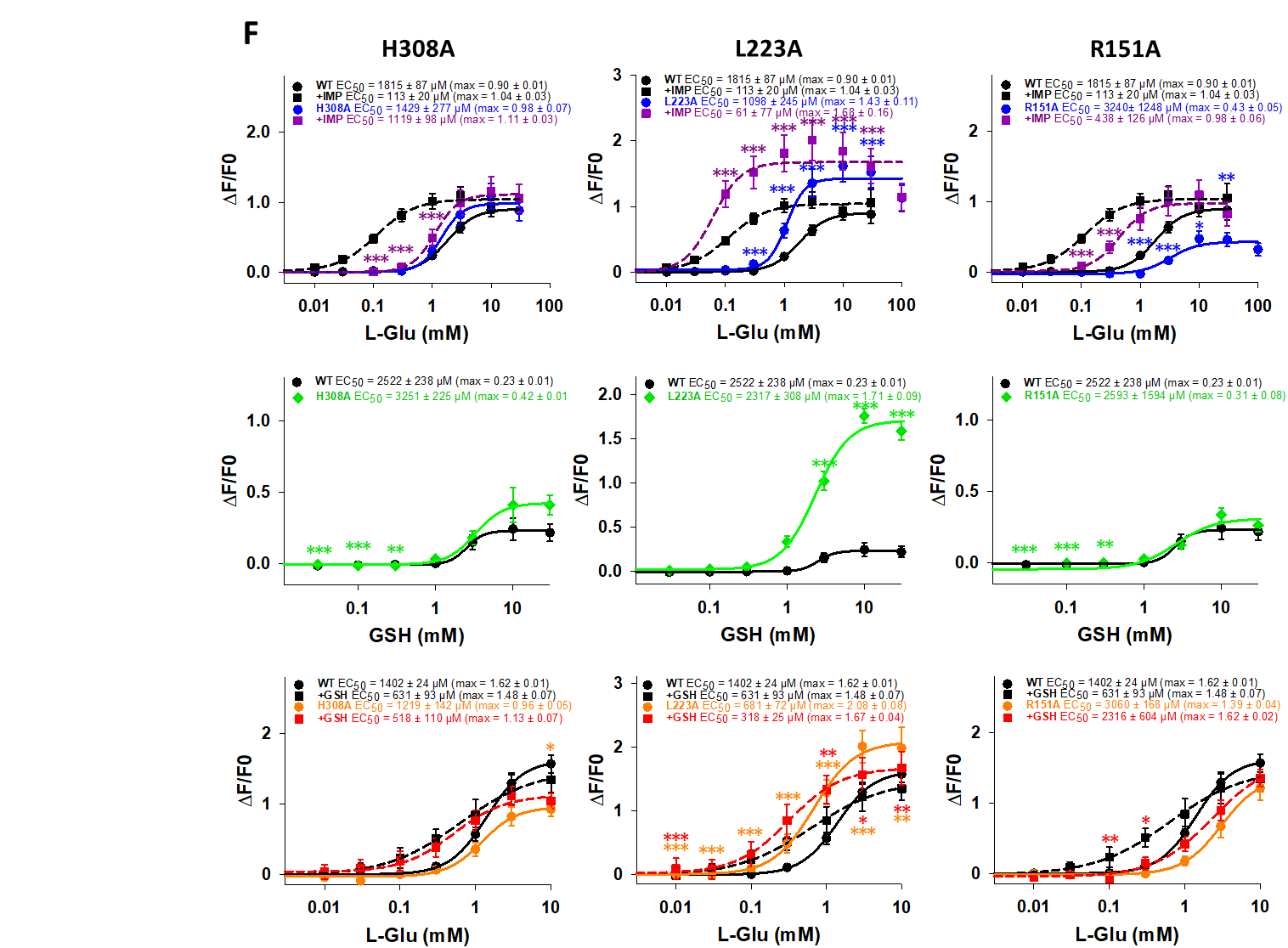

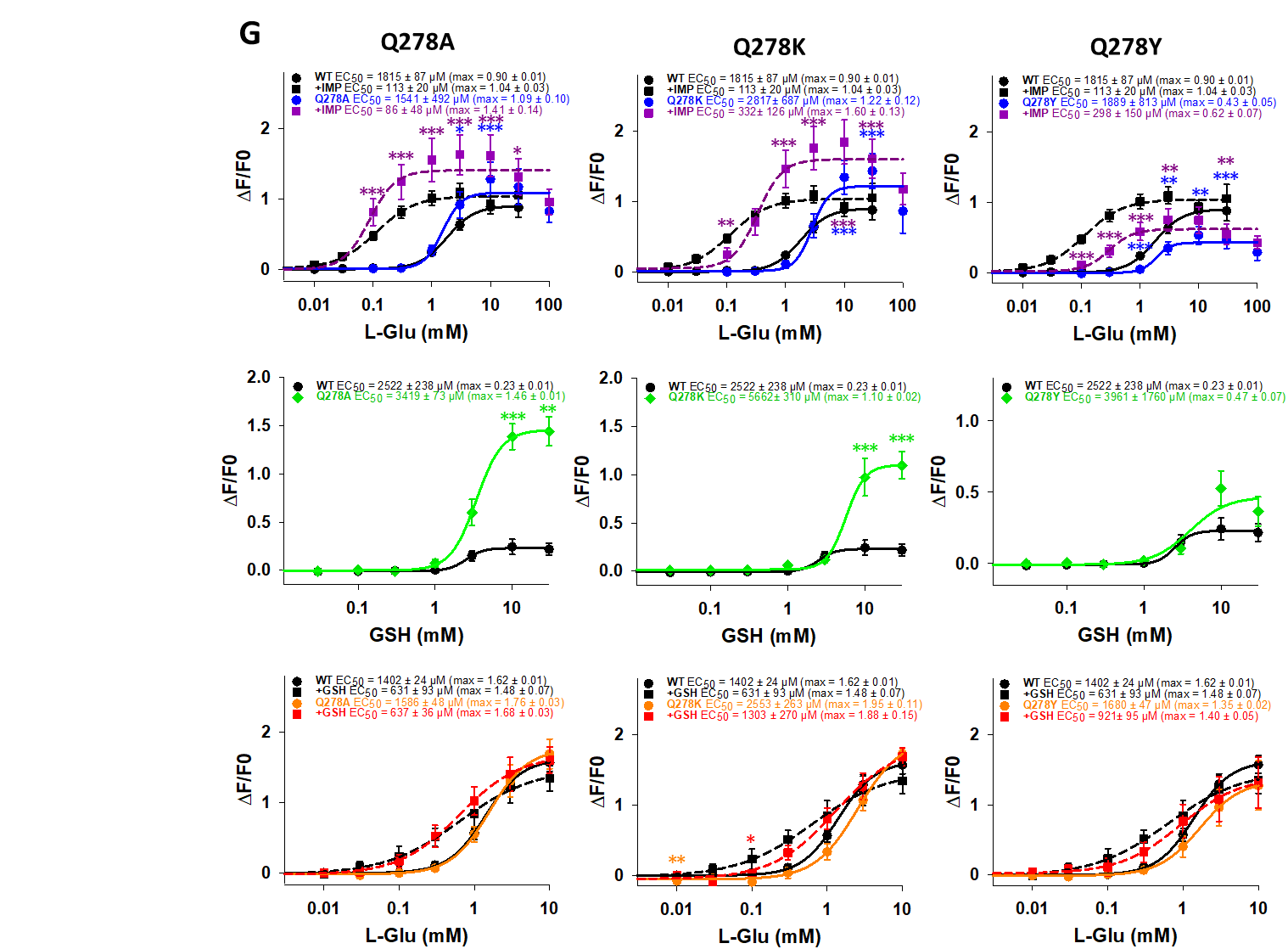


**Figure S4.** Dose-response curves of TAS1R1-WT (black curves) or TAS1R1 mutants (colored curves) co-expressed with TAS1R3 and stimulated with L-Glu (black and blue full curves) and with L-Glu + 1 mM IMP (black and purple dotted curves); GSH (black and green full curves); L-Glu (black and orange full curves); and L-Glu + 1 mM GSH (black and red dotted curves). The mutants are as follows: S172A, D192A, D192F (A); D192E, Y220A, E301A (B); D147A, A170W, A302W (C); H71A, H71Y, H71S (D); H71L, R277A, S306 (E); H308A, L223A, R151A (F); and Q278A, Q278K, Q278Y (G). The data are presented as the mean ± sem of 8 wells from 4 independent experiments. * *p* < 0.05, ** *p* < 0.01, *** *p* < 0.001, calculated via ANOVA followed by Dunnett’s test (with reference to TAS1R1-WT/TAS1R3-WT). The *p*-values are presented in Table S3. WT: wild-type; L-Glu: L-glutamic acid; IMP: inosine 5’-monophosphate, GSH: reduced L-glutathione; nd: not determined.


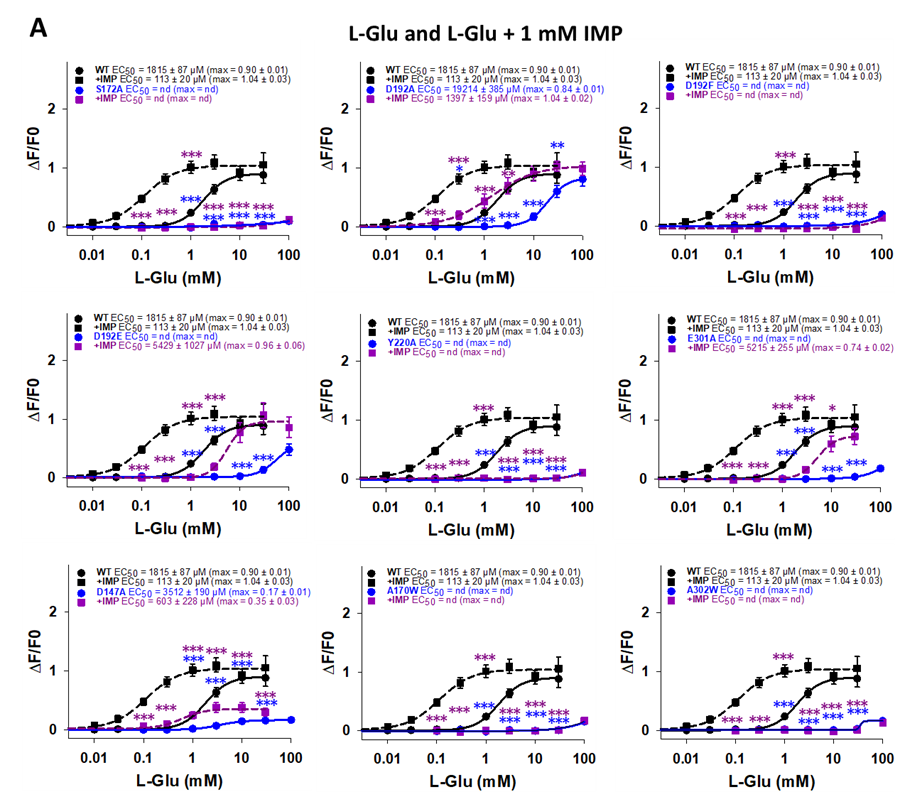

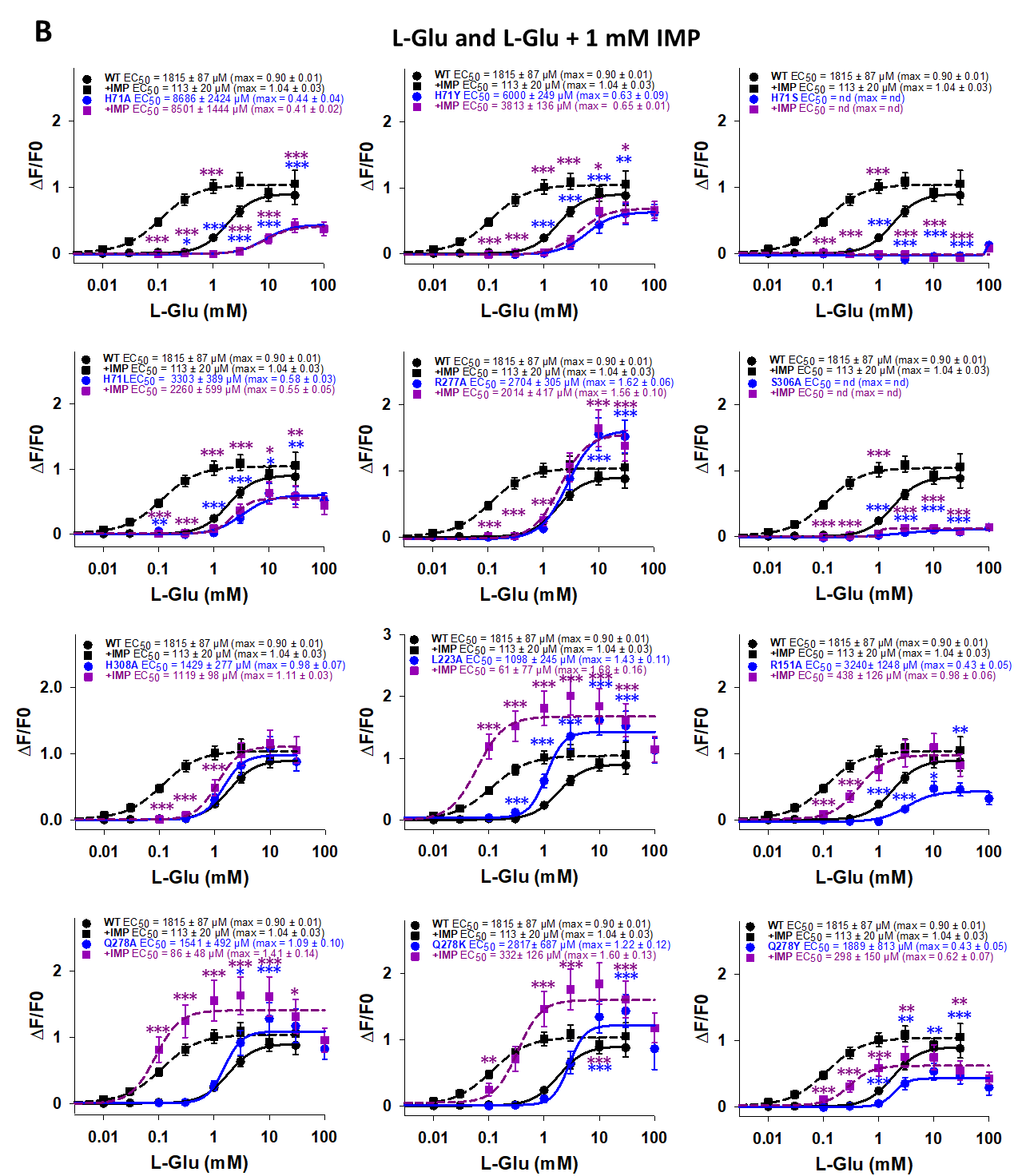


**Figure S5.** Dose-response curves of TAS1R1-WT (black curves) or TAS1R1 mutants (blue and purple curves) co-expressed with TAS1R3 and stimulated with L-Glu (black and blue full curves) or L-Glu + 1 mM IMP (black and purple dotted curves). The mutants are as follows: S172A, D192A, D192F, D192E, Y220A, E301A, D147A, A170W, A302W (A) and H71A, H71Y, H71S, H71L, R277A, S306A, H308A, L223A, R151A, Q278A, Q278K, Q278Y (B). The data are presented as the mean ± sem of 8 wells from 4 independent experiments. * *p* < 0.05, ** *p* < 0.01, *** *p* < 0.001, calculated via ANOVA followed by Dunnett’s test (with reference to TAS1R1-WT/TAS1R3-WT). The *p*-values are presented in Table S3. WT: wild-type; L-Glu: L-glutamic acid; IMP: inosine 5’-monophosphate; nd: not determined.


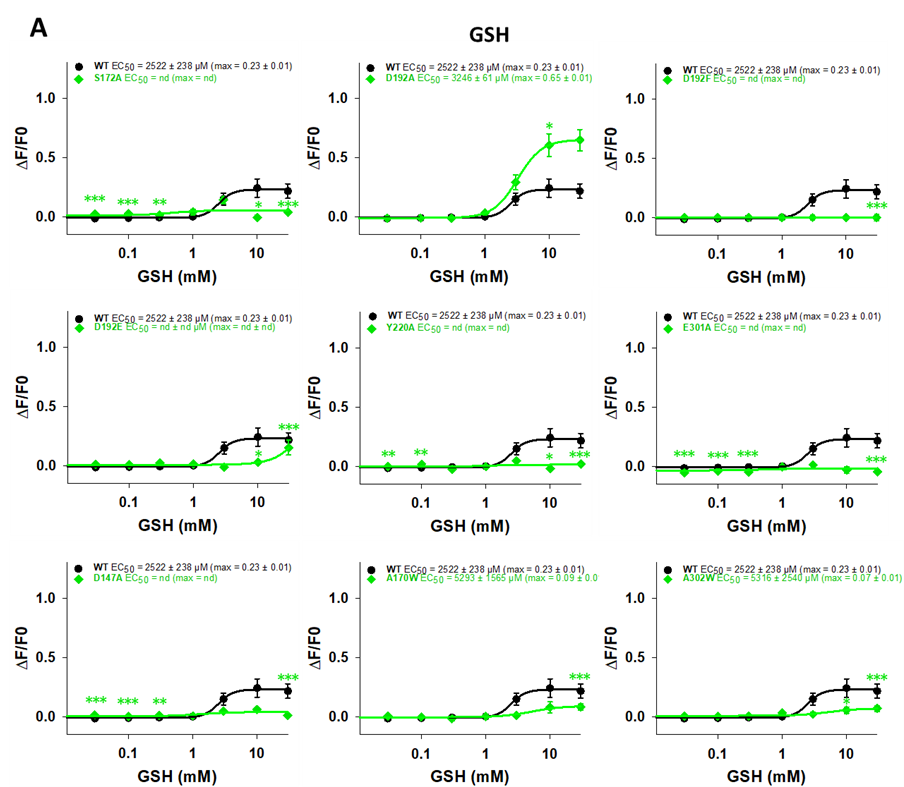

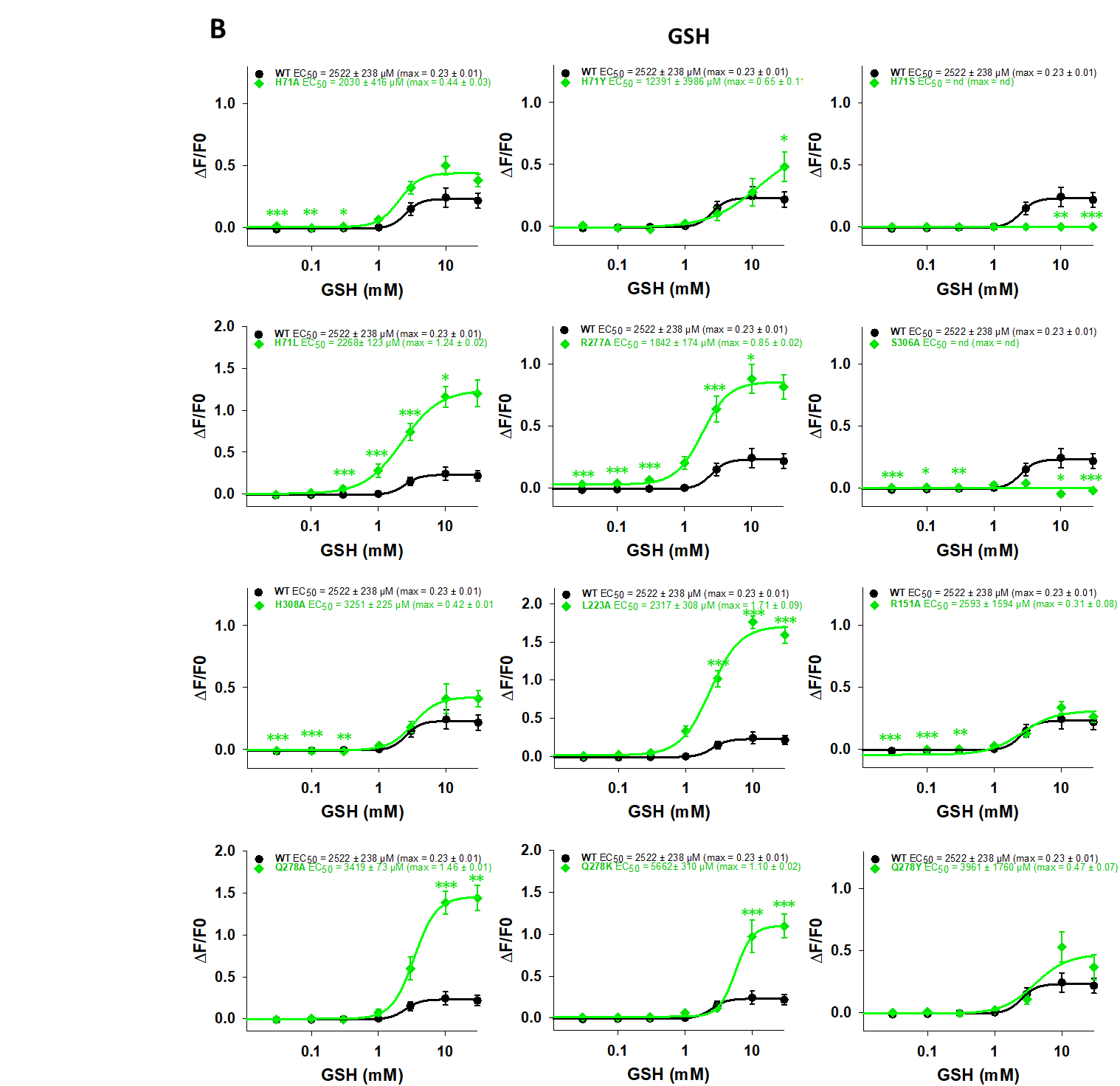


**Figure S6.** Dose-response curves of TAS1R1-WT (black curves) or TAS1R1 mutants (green curves) co-expressed with TAS1R3 and stimulated with GSH. The mutants are as follows: S172A, D192A, D192F, D192E, Y220A, E301A, D147A, A170W, and A302W (A) and H71A, H71Y, H71S, H71L, R277A, S306A, H308A, L223A, R151A, Q278A, Q278K, and Q278Y (B). The data are presented as the mean ± sem of 8 wells from 4 independent experiments. * *p* < 0.05, ** *p* < 0.01, *** *p* < 0.001, calculated via ANOVA followed by Dunnett’s test (with reference to TAS1R1-WT/TAS1R3-WT). The *p*-values are presented in Table S3. WT: wild-type; GSH: reduced L-glutathione; nd: not determined.


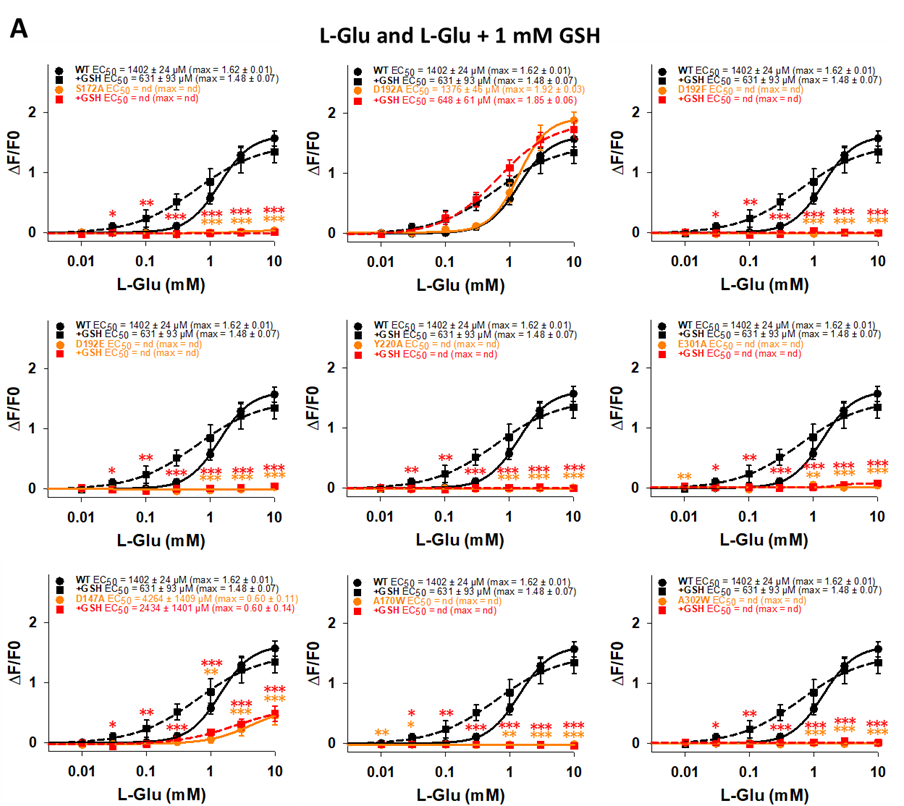

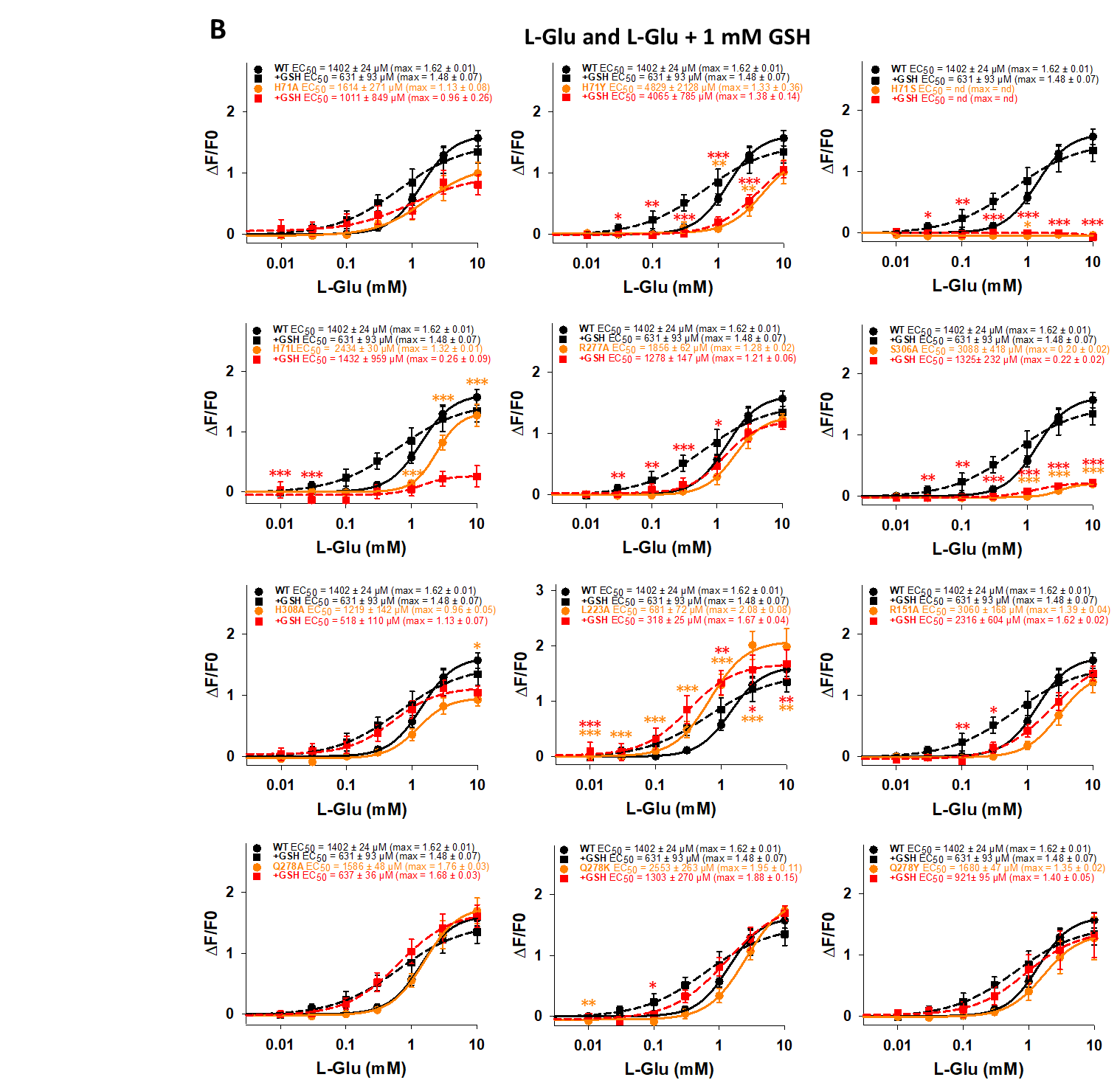


**Figure S7.** Dose-response curves of TAS1R1-WT (black curves) or TAS1R1 mutants (orange and red curves) co-expressed with TAS1R3 and stimulated with L-Glu (black and orange full curves) or L-Glu + 1 mM GSH (black and red dotted curves). The mutants are as follows: S172A, D192A, D192F, D192E, Y220A, E301A, D147A, A170W, and A302W (A) and H71A, H71Y, H71S, H71L, R277A, S306A, H308A, L223A, R151A, Q278A, Q278K, and Q278Y (B). The data are presented as the mean ± sem of 8 wells from 4 independent experiments. * *p* < 0.05, ** *p* < 0.01, *** *p* < 0.001, calculated via ANOVA followed by Dunnett’s test (with reference to TAS1R1-WT/TAS1R3-WT). The *p*-values are presented in Table S3. WT: wild-type; L-Glu: L-glutamic acid; GSH: reduced L-glutathione; nd: not determined.


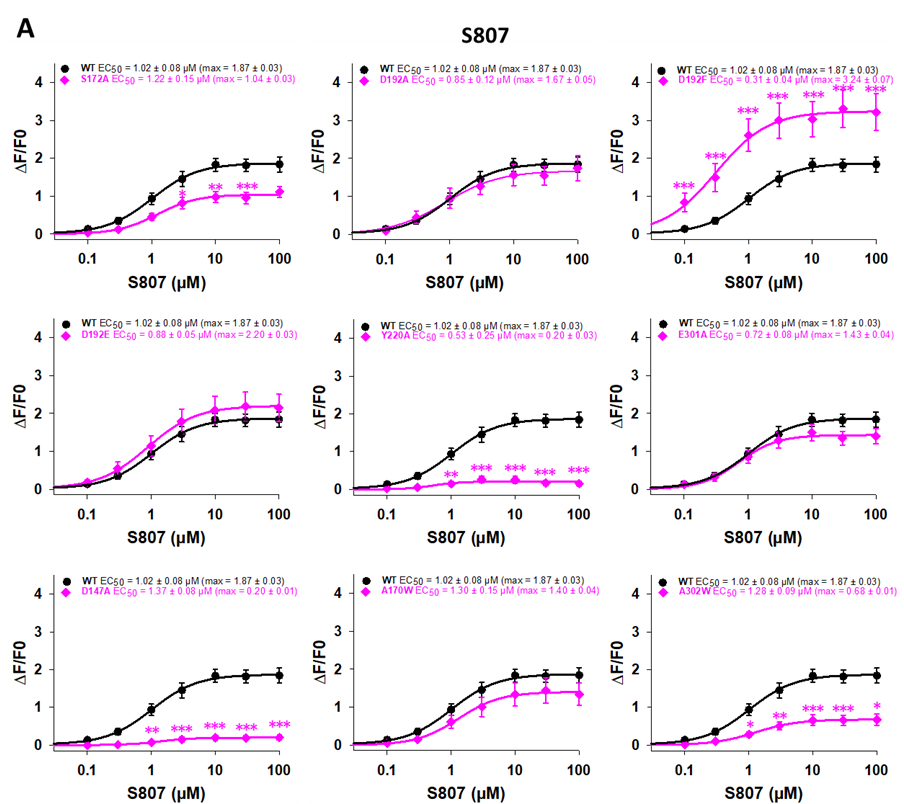

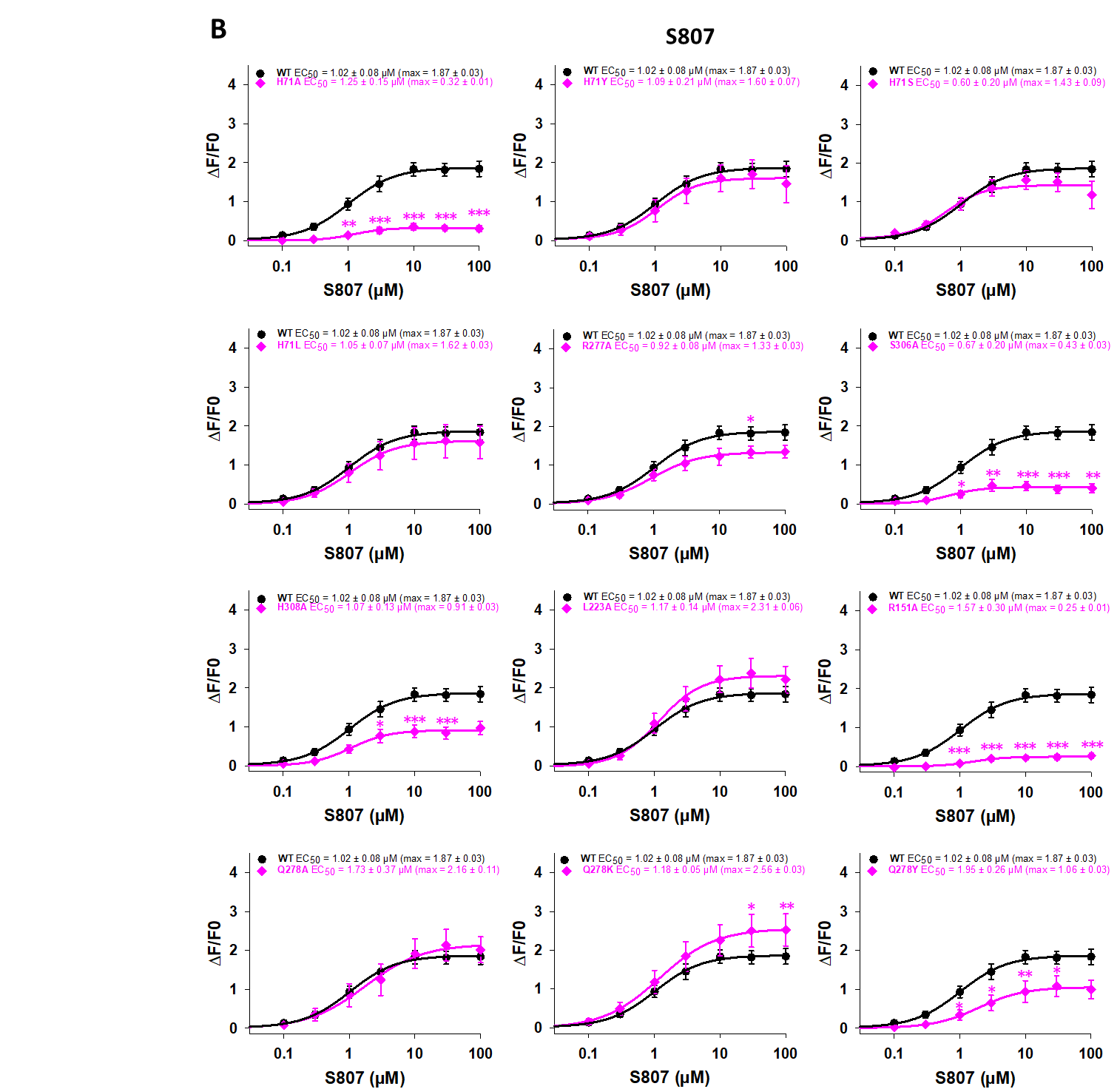


**Figure S8.** Dose-response curves of TAS1R1-WT (black curves) or TAS1R1 mutants (pink curves) co-expressed with TAS1R3 and stimulated with S807. The mutants are as follows: S172A, D192A, D192F, D192E, Y220A, E301A, D147A, A170W, and A302W (A) and H71A, H71Y, H71S, H71L, R277A, S306A, H308A, L223A, R151A, Q278A, Q278K, and Q278Y (B). The data are presented as the mean ± sem of 8 wells from 4 independent experiments. * *p* < 0.05, ** *p* < 0.01, *** *p* < 0.001, calculated via ANOVA followed by Dunnett’s test (with reference to TAS1R1-WT/TAS1R3-WT). The *p*-values are presented in Table S3. WT: wild-type; S807: N-(heptan-4-yl)benzo[d][1,3]dioxole-5-carboxamide.

**Table S1.** p-Values calculated using ANOVA followed by Dunnett’s test for multiple comparison analysis of ΔF/F0 for each compound or mix concentration and each rTAS1R3 mutants co-expressed with hTAS1R2 (with reference to hTAS1R2-WT/rTAS1R3-WT and hTAS1R1-WT/rTAS1R3-WT for sucralose (with or without GSH) and L-Glu (with and without IMP), GSH, L-Glu (with and without GSH) and S807, respectively). Significative p-value are in bold (p < 0.05).

|  |  | Concentration | rTAS1R3(Mut1) | rTAS1R3(Mut2) | rTAS1R3(Mut3) |
| --- | --- | --- | --- | --- | --- |
| hTAS1R2/rTAS1R3 | **Sucralose (mM)** | 0.01 | 0.201 | 0.959 | 0.219 |
|  |  | 0.03 | 0.833 | 1.000 | 0.168 |
|  |  | 0.1 | **0.001** | 0.069 | 0.849 |
|  |  | 0.3 | **< 0.0001** | **0.001** | 0.412 |
|  |  | 1 | **< 0.0001** | **0.001** | 0.494 |
|  |  | 3 | 0.248 | 0.466 | 0.951 |
|  |  | 10 | 0.110 | 0.290 | 0.868 |
|  | **Sucralose (mM) + 10 mM GSH** | 0.01 | **< 0.0001** | **< 0.0001** | **< 0.0001** |
|  |  | 0.03 | **< 0.0001** | **< 0.0001** | **< 0.0001** |
|  |  | 0.1 | 0.062 | **< 0.0001** | **0.001** |
|  |  | 0.3 | 0.974 | **0.008** | 0.097 |
|  |  | 1 | 0.420 | 0.956 | 0.589 |
|  |  | 3 | 0.851 | 0.997 | 0.940 |
|  |  | 10 | 0.945 | 0.789 | 0.992 |
| hTAS1R1/rTAS1R3 | **L-Glu (mM)**  **(IMP serie)** | 0.01 | **0.027** | 0.949 | **0.042** |
|  |  | 0.03 | 0.112 | 0.999 | 0.112 |
|  |  | 0.1 | **0.017** | 0.998 | **0.018** |
|  |  | 0.3 | **0.0001** | 0.643 | **0.001** |
|  |  | 1 | **0.002** | **0.0004** | **< 0.0001** |
|  |  | 3 | 0.096 | **< 0.0001** | **< 0.0001** |
|  |  | 10 | 0.060 | **< 0.0001** | **< 0.0001** |
|  | **L-Glu (mM) + 1 mM IMP** | 0.01 | 0.136 | **0.011** | **0.001** |
|  |  | 0.03 | 0.314 | **0.004** | **0.001** |
|  |  | 0.1 | 0.329 | **< 0.0001** | **< 0.0001** |
|  |  | 0.3 | 0.447 | **< 0.0001** | **< 0.0001** |
|  |  | 1 | 0.457 | **< 0.0001** | **< 0.0001** |
|  |  | 3 | 0.243 | **< 0.0001** | **< 0.0001** |
|  |  | 10 | 0.079 | **< 0.0001** | **< 0.0001** |
|  | **GSH (mM)** | 0.03 | **< 0.0001** | **< 0.0001** | **< 0.0001** |
|  |  | 0.1 | **0.0003** | **< 0.0001** | **< 0.0001** |
|  |  | 0.3 | **< 0.0001** | **< 0.0001** | **< 0.0001** |
|  |  | 1 | **< 0.0001** | **< 0.0001** | **< 0.0001** |
|  |  | 3 | **< 0.0001** | **< 0.0001** | **< 0.0001** |
|  |  | 10 | **< 0.0001** | **< 0.0001** | **< 0.0001** |
|  |  | 30 | **0.001** | **< 0.0001** | **< 0.0001** |
|  | **L-Glu (mM)**  **(GSH serie)** | 0.01 | 1.000 | **< 0.0001** | **< 0.0001** |
|  |  | 0.03 | **0.009** | **< 0.0001** | **< 0.0001** |
|  |  | 0.1 | **0.002** | **< 0.0001** | **< 0.0001** |
|  |  | 0.3 | **0.005** | **< 0.0001** | **< 0.0001** |
|  |  | 1 | **0.002** | **< 0.0001** | **< 0.0001** |
|  |  | 3 | 0.183 | **< 0.0001** | **< 0.0001** |
|  |  | 10 | 1.000 | **< 0.0001** | **< 0.0001** |
|  | **L-Glu (mM) + 10 mM GSH** | 0.01 | 0.184 | **< 0.0001** | **< 0.0001** |
|  |  | 0.03 | **0.024** | **< 0.0001** | **< 0.0001** |
|  |  | 0.1 | **0.0004** | **< 0.0001** | **< 0.0001** |
|  |  | 0.3 | 0.220 | **< 0.0001** | **< 0.0001** |
|  |  | 1 | **0.030** | **< 0.0001** | **< 0.0001** |
|  |  | 3 | 0.988 | **0.034** | **0.032** |
|  |  | 10 | 0.997 | **< 0.0001** | **< 0.0001** |
|  | **S807 (µM)** | 0.1 | 0.088 | **0.003** | **0.010** |
|  |  | 0.3 | 0.244 | **0.001** | **0.004** |
|  |  | 1 | 0.102 | **0.004** | **0.022** |
|  |  | 3 | 0.181 | **0.0005** | **0.010** |
|  |  | 10 | 0.233 | **0.0001** | **0.009** |
|  |  | 30 | 0.377 | **< 0.0001** | **0.004** |
|  |  | 100 | 0.301 | **< 0.0001** | **0.001** |

WT: wild-type; L-Glu: L-glutamic acid; IMP: inosine 5’-monophosphate; GSH: reduced L-glutathione; S807: N-(heptan-4-yl)benzo[d][1,3]dioxole-5-carboxamide.

**Table S2**. p-Values calculated using ANOVA followed by Dunnett’s test for multiple comparison analysis of ΔF/F0 for each mix concentration with reference to L-Glu alone for IMP, GMP, and GSH, and to GSH alone for IMP, GMP and L-Glu. Significative p-value are in bold (p < 0.05).

|  | Concentration | + 10 µM IMP | + 100 µM IMP | + 1000 µM IMP |
| --- | --- | --- | --- | --- |
| L-Glu (mM) | 0.01 | 0.360 | 0.310 | **0.0004** |
|  | 0.03 | 0.988 | 0.999 | **< 0.0001** |
|  | 0.1 | 0.977 | 0.726 | **< 0.0001** |
|  | 0.3 | 0.999 | 0.076 | **< 0.0001** |
|  | 1 | 0.963 | **0.001** | **< 0.0001** |
|  | 3 | 0.996 | 0.488 | 0.051 |
|  | 10 | 0.999 | 0.590 | 0.333 |
|  | Concentration | **+ 10 µM GMP** | **+ 100 µM GMP** | **+ 1000 µM GMP** |
| L-Glu (mM) | 0.01 | 0.724 | 0.995 | **0.001** |
|  | 0.03 | 0.920 | 0.823 | **< 0.0001** |
|  | 0.1 | 0.986 | **0.011** | **< 0.0001** |
|  | 0.3 | 0.985 | **< 0.0001** | **< 0.0001** |
|  | 1 | 0.458 | **0.004** | **< 0.0001** |
|  | 3 | 0.938 | 0.842 | 0.225 |
|  | 10 | 0.630 | 0.980 | 1.000 |
|  | Concentration | **+ 1 mM IMP** | **+ 3 mM IMP** | **+ 10 mM IMP** |
| GSH (mM) | 0.03 | **0.028** | **0.013** | 0.122 |
|  | 0.1 | **< 0.0001** | **0.0004** | **0.001** |
|  | 0.3 | **0.012** | **0.002** | **< 0.0001** |
|  | 1 | 0.149 | 0.143 | 0.070 |
|  | 3 | 0.852 | 0.545 | 1.000 |
|  | 10 | 1.000 | 1.000 | 0.672 |
|  | 30 | 1.000 | 1.000 | 0.724 |
|  | Concentration | **+ 1 mM GMP** | **+ 3 mM GMP** | **+ 10 mM GMP** |
| GSH (mM) | 0.03 | 0.999 | 0.344 | 0.075 |
|  | 0.1 | 0.785 | 1.000 | 0.399 |
|  | 0.3 | 0.633 | **0.001** | **0.0003** |
|  | 1 | 0.295 | **0.006** | **0.0004** |
|  | 3 | 0.462 | 0.130 | 0.067 |
|  | 10 | 0.987 | 0.761 | 0.996 |
|  | 30 | 1.000 | 1.000 | 0.366 |
|  | Concentration | **+ 100 µM L-Glu** | **+ 300 µM L-Glu** | **+ 1000 µM L-Glu** |
| GSH (mM) | 0.03 | 0.969 | **0.002** | **< 0.0001** |
|  | 0.1 | 0.949 | **< 0.0001** | **< 0.0001** |
|  | 0.3 | 0.452 | **< 0.0001** | **< 0.0001** |
|  | 1 | 0.177 | **< 0.0001** | **< 0.0001** |
|  | 3 | 0.178 | **0.0001** | **< 0.0001** |
|  | 10 | 0.753 | 0.151 | **0.012** |
|  | 30 | 0.202 | 0.148 | 0.134 |
|  | Concentration | **+ 1 mM GSH** | **+ 3 mM GSH** | **+ 10 mM GSH** |
| L-Glu (mM) | 0.03 | 0.605 | **0.004** | **< 0.0001** |
|  | 0.1 | 0.226 | **0.002** | **< 0.0001** |
|  | 0.3 | 0.120 | **0.001** | **< 0.0001** |
|  | 1 | 0.107 | **0.006** | **< 0.0001** |
|  | 3 | 0.189 | **0.021** | **0.005** |
|  | 10 | 0.906 | 0.998 | 0.999 |
|  | 30 | 0.986 | 0.999 | 0.796 |

L-Glu: L-glutamic acid; IMP: inosine 5’-monophosphate; GMP: guanosine 5’-monophosphate; GSH: reduced L-glutathione.

**Table S3**. p-Values calculated using ANOVA followed by Dunnett’s test for multiple comparison analysis of ΔF/F0 for each compound or mix concentration and each TAS1R3 mutants co-expressed with TAS1R3 (with reference to TAS1R1-WT/TAS1R3-WT). Significative p-value are in bold (p < 0.05).

|  |  | Mutants | | | | | | | | | | | | | | | | | | | | |
| --- | --- | --- | --- | --- | --- | --- | --- | --- | --- | --- | --- | --- | --- | --- | --- | --- | --- | --- | --- | --- | --- | --- |
|  | **Concentration** | **S172A** | **D192A** | **D192F** | **D192E** | **Y220A** | **E301A** | **D147A** | **A170W** | **A302W** | **H71A** | **H71Y** | **H71S** | **H71L** | **R277A** | **S306A** | **H308A** | **L223A** | **R151A** | **Q278A** | **Q278K** | **Q278Y** |
| L-Glu (IMP serie) | 0.1 | 1.000 | 0.541 | 0.952 | 0.953 | 1.000 | 1.000 | 1.000 | 0.996 | 1.000 | 1.000 | 1.000 | 0.930 | **0.0004** | 1.000 | 1.000 | 0.996 | 0.122 | 1.000 | 0.323 | 0.998 | 0.959 |
|  | 0.3 | 0.426 | **0.020** | 1.000 | 0.997 | 0.266 | 0.992 | 0.499 | 1.000 | 0.709 | **0.044** | 1.000 | 1.000 | 1.000 | 1.000 | 1.000 | 0.975 | **< 0.0001** | 0.449 | 0.413 | 1.000 | 1.000 |
|  | 1 | **< 0.0001** | **< 0.0001** | **< 0.0001** | **< 0.0001** | **< 0.0001** | **< 0.0001** | **< 0.0001** | **< 0.0001** | **< 0.0001** | **< 0.0001** | **< 0.0001** | **< 0.0001** | **< 0.0001** | 0.054 | **< 0.0001** | 0.717 | **< 0.0001** | **< 0.0001** | 0.534 | 0.070 | **0.0002** |
|  | 3 | **< 0.0001** | **< 0.0001** | **< 0.0001** | **< 0.0001** | **< 0.0001** | **< 0.0001** | **< 0.0001** | **< 0.0001** | **< 0.0001** | **< 0.0001** | **< 0.0001** | **< 0.0001** | **< 0.0001** | 0.087 | **< 0.0001** | 0.705 | **< 0.0001** | **< 0.0001** | **0.013** | 0.949 | 1.000 |
|  | 10 | **< 0.0001** | **< 0.0001** | **< 0.0001** | **< 0.0001** | **< 0.0001** | **< 0.0001** | **< 0.0001** | **< 0.0001** | **< 0.0001** | **< 0.0001** | **< 0.0001** | **< 0.0001** | **0.024** | **< 0.0001** | **< 0.0001** | 0.695 | **< 0.0001** | **0.024** | **0.0004** | **< 0.0001** | **0.001** |
|  | 30 | **< 0.0001** | **0.003** | **< 0.0001** | **< 0.0001** | **< 0.0001** | **< 0.0001** | **< 0.0001** | **< 0.0001** | **< 0.0001** | **< 0.0001** | **0.003** | **< 0.0001** | **0.001** | **< 0.0001** | **< 0.0001** | 0.999 | **< 0.0001** | **0.002** | 0.096 | **< 0.0001** | **< 0.0001** |
| L-Glu (mM)+ 1 mM IMP | 0.1 | **< 0.0001** | **< 0.0001** | **< 0.0001** | **< 0.0001** | **< 0.0001** | **< 0.0001** | **< 0.0001** | **< 0.0001** | **< 0.0001** | **< 0.0001** | **< 0.0001** | **< 0.0001** | **< 0.0001** | **< 0.0001** | **< 0.0001** | **< 0.0001** | **< 0.0001** | **< 0.0001** | **0.0001** | **0.002** | **< 0.0001** |
|  | 0.3 | **< 0.0001** | **< 0.0001** | **< 0.0001** | **< 0.0001** | **< 0.0001** | **< 0.0001** | **< 0.0001** | **< 0.0001** | **< 0.0001** | **< 0.0001** | **< 0.0001** | **< 0.0001** | **< 0.0001** | **< 0.0001** | **< 0.0001** | **< 0.0001** | **< 0.0001** | **< 0.0001** | **< 0.0001** | 0.986 | **< 0.0001** |
|  | 1 | **< 0.0001** | **< 0.0001** | **< 0.0001** | **< 0.0001** | **< 0.0001** | **< 0.0001** | **< 0.0001** | **< 0.0001** | **< 0.0001** | **< 0.0001** | **< 0.0001** | **< 0.0001** | **< 0.0001** | **< 0.0001** | **< 0.0001** | **< 0.0001** | **< 0.0001** | 0.860 | **< 0.0001** | **0.0001** | **0.0002** |
|  | 3 | **< 0.0001** | **0.003** | **< 0.0001** | **< 0.0001** | **< 0.0001** | **< 0.0001** | **< 0.0001** | **< 0.0001** | **< 0.0001** | **< 0.0001** | **< 0.0001** | **< 0.0001** | **< 0.0001** | 1.000 | **< 0.0001** | 0.956 | **< 0.0001** | 1.000 | **< 0.0001** | **< 0.0001** | **0.004** |
|  | 10 | **< 0.0001** | 0.288 | **< 0.0001** | 0.712 | **< 0.0001** | **0.014** | **< 0.0001** | **< 0.0001** | **< 0.0001** | **< 0.0001** | **0.039** | **< 0.0001** | **0.016** | **< 0.0001** | **< 0.0001** | 0.975 | **< 0.0001** | 0.400 | **< 0.0001** | **< 0.0001** | 0.322 |
|  | 30 | **< 0.0001** | 0.954 | **< 0.0001** | 0.998 | **< 0.0001** | 0.322 | **< 0.0001** | **< 0.0001** | **< 0.0001** | **< 0.0001** | **0.014** | **< 0.0001** | **0.002** | **0.005** | **< 0.0001** | 1.000 | **< 0.0001** | 1.000 | **0.013** | **< 0.0001** | **0.001** |
| GSH (mM) | 0.03 | **< 0.0001** | 0.377 | 1.000 | 1.000 | **0.001** | **< 0.0001** | **< 0.0001** | 1.000 | 0.999 | **< 0.0001** | 1.000 | 0.998 | 0.989 | **< 0.0001** | **< 0.0001** | **< 0.0001** | 1.000 | **< 0.0001** | 0.768 | 1.000 | 1.000 |
|  | 0.1 | **< 0.0001** | 0.948 | 0.996 | 0.982 | **0.004** | **< 0.0001** | **0.0004** | 0.999 | 0.530 | **0.001** | 1.000 | 1.000 | 1.000 | **< 0.0001** | **< 0.0001** | **0.0001** | 0.998 | **< 0.0001** | 0.957 | 0.942 | 0.992 |
|  | 0.3 | **0.001** | 0.965 | 1.000 | 1.000 | 0.435 | **< 0.0001** | **0.003** | 1.000 | 0.992 | **0.034** | 1.000 | 1.000 | **< 0.0001** | **< 0.0001** | **0.004** | **0.001** | 1.000 | **0.008** | 1.000 | 1.000 | 0.998 |
|  | 1 | 0.177 | 0.815 | 1.000 | 1.000 | 0.936 | 0.112 | 0.611 | 1.000 | 1.000 | 0.155 | 1.000 | 1.000 | **< 0.0001** | **< 0.0001** | 0.075 | 0.168 | 0.059 | 0.160 | 1.000 | 0.999 | 1.000 |
|  | 3 | 1.000 | 0.078 | 0.314 | 0.119 | 0.557 | 0.971 | 0.870 | 0.098 | 0.117 | 0.789 | 0.907 | 0.145 | **< 0.0001** | **< 0.0001** | 0.919 | 1.000 | **< 0.0001** | 1.000 | 1.000 | 0.890 | 0.907 |
|  | 10 | **0.018** | **0.030** | 0.800 | **0.010** | **0.010** | 0.053 | 0.090 | 0.051 | **0.024** | 1.000 | 0.967 | **0.004** | **0.041** | **0.030** | **0.015** | 1.000 | **< 0.0001** | 1.000 | **< 0.0001** | **0.0004** | 1.000 |
|  | 30 | **< 0.0001** | 0.109 | **< 0.0001** | **0.0002** | **< 0.0001** | **< 0.0001** | **< 0.0001** | **< 0.0001** | **< 0.0001** | 0.376 | 0.987 | **< 0.0001** | 1.000 | 0.896 | **< 0.0001** | 0.729 | **< 0.0001** | 0.054 | **0.007** | **< 0.0001** | 0.270 |
| L-Glu (mM) (GSH serie) | 0.01 | 0.531 | 0.896 | 0.399 | 0.536 | 0.997 | **0.004** | 0.821 | **0.001** | 0.441 | 1.000 | 0.784 | 0.663 | 0.094 | 0.508 | 0.763 | 0.646 | **0.0005** | 0.873 | 0.094 | **0.018** | 0.973 |
|  | 0.03 | 0.961 | 0.989 | 0.986 | 0.881 | 1.000 | 0.168 | 0.355 | **0.011** | 0.916 | 1.000 | 1.000 | 0.439 | 0.969 | 0.152 | 1.000 | 0.848 | **0.0004** | 0.995 | 0.599 | 0.193 | 1.000 |
|  | 0.1 | 1.000 | 0.326 | 1.000 | 0.999 | 1.000 | 1.000 | 1.000 | 0.905 | 1.000 | 0.977 | 1.000 | 1.000 | 1.000 | 0.526 | 0.744 | 0.900 | **< 0.0001** | 1.000 | 0.993 | 1.000 | 1.000 |
|  | 0.3 | 0.164 | 1.000 | 0.248 | 0.141 | 0.105 | 0.543 | 0.884 | 0.587 | 0.292 | 1.000 | 1.000 | 0.481 | 0.449 | 0.280 | 0.106 | 0.853 | **< 0.0001** | 0.472 | 1.000 | 1.000 | 0.972 |
|  | 1 | **0.0003** | 1.000 | **0.0002** | **0.0001** | **0.0001** | **0.002** | **0.004** | **0.001** | **0.0002** | 0.592 | **0.002** | **0.033** | **0.0004** | 0.054 | **0.0002** | 0.515 | **< 0.0001** | 0.056 | 1.000 | 1.000 | 0.967 |
|  | 3 | **< 0.0001** | 0.317 | **< 0.0001** | **< 0.0001** | **< 0.0001** | **< 0.0001** | **< 0.0001** | **< 0.0001** | **< 0.0001** | 0.125 | **0.003** | 0.728 | **< 0.0001** | 0.659 | **< 0.0001** | 0.396 | **< 0.0001** | 0.186 | 1.000 | 1.000 | 0.994 |
|  | 10 | **< 0.0001** | 0.055 | **< 0.0001** | **< 0.0001** | **< 0.0001** | **< 0.0001** | **< 0.0001** | **< 0.0001** | **< 0.0001** | 0.178 | 0.513 | 1.000 | **< 0.0001** | 0.988 | **< 0.0001** | **0.049** | **0.005** | 1.000 | 0.556 | 0.082 | 1.000 |
| L-Glu (mM) + 1 mM GSH | 0.01 | 0.472 | 1.000 | 0.529 | 0.462 | 0.372 | 0.703 | 0.654 | 0.729 | 0.521 | 0.476 | 0.457 | 0.701 | **< 0.0001** | 0.372 | 0.202 | 0.998 | **0.015** | 0.950 | 1.000 | 0.939 | 1.000 |
|  | 0.03 | **0.031** | 0.872 | **0.013** | **0.014** | 0**.008** | **0.037** | **0.048** | **0.046** | **0.028** | 1.000 | **0.040** | **0.034** | **< 0.0001** | **0.006** | **0.008** | 0.525 | 0.710 | 0.317 | 0.689 | 0.312 | 0.579 |
|  | 0.1 | **0.002** | 0.973 | **0.001** | **0.001** | **0.001** | **0.004** | **0.003** | **0.005** | **0.001** | **0.992** | **0.002** | **0.002** | 0.055 | **0.002** | **0.001** | 0.155 | 0.992 | **0.006** | 0.568 | **0.017** | 0.099 |
|  | 0.3 | **< 0.0001** | 1.000 | **< 0.0001** | **< 0.0001** | **< 0.0001** | **< 0.0001** | **0.003** | **0.0001** | **< 0.0001** | 0.794 | **< 0.0001** | **< 0.0001** | 0.577 | **0.0002** | **< 0.0001** | 0.130 | 0.084 | **0.014** | 0.999 | 0.229 | 0.191 |
|  | 1 | **< 0.0001** | 0.998 | **< 0.0001** | **< 0.0001** | **< 0.0001** | **< 0.0001** | **< 0.0001** | **< 0.0001** | **< 0.0001** | 0.155 | **< 0.0001** | **< 0.0001** | 1.000 | **0.003** | **< 0.0001** | 0.892 | **0.002** | 0.074 | 1.000 | 0.998 | 0.984 |
|  | 3 | **< 0.0001** | 0.860 | **< 0.0001** | **< 0.0001** | **< 0.0001** | **< 0.0001** | **< 0.0001** | **< 0.0001** | **< 0.0001** | 0.717 | **0.0003** | **< 0.0001** | 1.000 | 0.298 | **< 0.0001** | 0.936 | **0.043** | 0.576 | 1.000 | 1.000 | 0.970 |
|  | 10 | **< 0.0001** | 0.108 | **< 0.0001** | **< 0.0001** | **< 0.0001** | **< 0.0001** | **< 0.0001** | **< 0.0001** | **< 0.0001** | 0.165 | 0.404 | **< 0.0001** | 1.000 | 0.693 | **< 0.0001** | 0.252 | **0.002** | 1.000 | 0.805 | 0.590 | 1.000 |
| S807 (µM) | 0.1 | 1.000 | 1.000 | **< 0.0001** | 1.000 | 1.000 | 0.993 | 0.859 | 0.613 | 0.481 | 0.864 | 1.000 | 0.996 | 0.991 | 1.000 | 1.000 | 1.000 | 0.602 | 0.351 | 0.996 | 1.000 | 0.432 |
|  | 0.3 | 0.823 | 0.999 | **< 0.0001** | 0.772 | 0.880 | 1.000 | 0.292 | 0.703 | 0.463 | 0.230 | 1.000 | 1.000 | 1.000 | 1.000 | 0.905 | 0.865 | 1.000 | 0.117 | 1.000 | 0.982 | 0.417 |
|  | 1 | 0.144 | 1.000 | **< 0.0001** | 0.988 | **0.006** | 1.000 | **0.001** | 0.651 | **0.021** | **0.001** | 1.000 | 1.000 | 1.000 | 0.988 | **0.044** | 0.107 | 1.000 | **0.0002** | 1.000 | 0.958 | **0.038** |
|  | 3 | **0.042** | 0.999 | **< 0.0001** | 0.709 | **< 0.0001** | 0.999 | **< 0.0001** | 0.528 | **0.002** | **< 0.0001** | 1.000 | 1.000 | 1.000 | 1.000 | **0.003** | **0.021** | 0.934 | **< 0.0001** | 1.000 | 0.523 | **0.011** |
|  | 10 | **0.001** | 0.935 | **< 0.0001** | 0.927 | **< 0.0001** | 0.573 | **< 0.0001** | 0.377 | **< 0.0001** | **< 0.0001** | 1.000 | 0.993 | 0.999 | 1.000 | **< 0.0001** | **0.0001** | 0.487 | **< 0.0001** | 1.000 | 0.364 | **0.003** |
|  | 30 | **0.0002** | 0.847 | **< 0.0001** | 0.564 | **< 0.0001** | 0.132 | **< 0.0001** | 0.621 | **< 0.0001** | **< 0.0001** | 1.000 | 0.952 | 1.000 | 1.000 | **< 0.0001** | **< 0.0001** | 0.088 | **< 0.0001** | 0.788 | **0.020** | **0.014** |
|  | 100 | 0.459 | 0.998 | **< 0.0001** | 0.131 | **< 0.0001** | 1.000 | **< 0.0001** | 1.000 | **0.029** | **< 0.0001** | 1.000 | 0.973 | 1.000 | 0.980 | **0.002** | 0.185 | 0.063 | **< 0.0001** | 0.370 | **0.002** | 0.452 |

WT: wild-type; L-Glu: L-glutamic acid; IMP: inosine 5’-monophosphate, GSH: reduced L-glutathione; S807: N-(heptan-4-yl)benzo[d][1,3]dioxole-5-carboxamide.
